# The long non-coding RNA NEAT1 is responsive to neuronal activity and is associated with hyperexcitability states

**DOI:** 10.1038/srep40127

**Published:** 2017-01-05

**Authors:** Guy Barry, James A. Briggs, Do Won Hwang, Sam P. Nayler, Patrick R. J. Fortuna, Nicky Jonkhout, Fabien Dachet, Jesper L. V. Maag, Pieter Mestdagh, Erin M. Singh, Lotta Avesson, Dominik C. Kaczorowski, Ezgi Ozturk, Nigel C. Jones, Irina Vetter, Luis Arriola-Martinez, Jianfei Hu, Gloria R. Franco, Victoria M. Warn, Andrew Gong, Marcel E. Dinger, Frank Rigo, Leonard Lipovich, Margaret J. Morris, Terence J. O’Brien, Dong Soo Lee, Jeffrey A. Loeb, Seth Blackshaw, John S. Mattick, Ernst J. Wolvetang

**Affiliations:** 1 QIMR Berghofer Medical Research Institute, Herston, QLD, Australia; 2 Garvan Institute of Medical Research, Sydney, New South Wales 2010, Australia; 3 Australian Institute for Bioengineering and Nanotechnology, The University of Queensland, Brisbane, Queensland, Australia; 4 Department of Nuclear Medicine, Seoul National University College of Medicine, Seoul, Republic of Korea; 5 Department of Molecular Medicine and Biopharmaceutical Sciences, Seoul National University, Seoul, Republic of Korea; 6 Department of Neurology and Rehabilitation, University of Illinois at Chicago, Chicago, IL, USA; 7 St Vincent’s Clinical School, UNSW Australia, Kensington NSW 2052, Australia; 8 Center for Medical Genetics, Ghent University 9000, Ghent, Belgium; 9 Department of Neuroscience, Neurology and Ophthalmology, Center for High-Throughput Biology and Institute for Cell Engineering, Johns Hopkins University School of Medicine, Baltimore, MD 21287, USA; 10 Department of Medicine, The University of Melbourne, Royal Melbourne Hospital, Parkville 3050, Victoria, Australia; 11 Institute for Molecular Bioscience, The University of Queensland, St. Lucia 4072, Queensland, Australia; 12 Wilmer Institute, Johns Hopkins University School of Medicine, Baltimore, MD 21287, USA; 13 Departamento de Bioquímica e Imunologia, Instituto de Ciências Biológicas, Universidade Federal de Minas Gerais Belo Horizonte, Brazil; 14 School of Medical Sciences, University of New South Wales, New South Wales 2052, Australia; 15 Kinghorn Centre for Clinical Genomics, Garvan Institute of Medical Research, Sydney, NSW, Australia; 16 Isis Pharmaceuticals, Carlsbad, CA 92010, USA; 17 Center for Molecular Medicine and Genetics, Wayne State University, Detroit, MI, USA; 18 Department of Neurology, Wayne State University School of Medicine, Detroit, USA; 19 Department of Pharmacology, University of New South Wales, New South Wales 2052, Australia

## Abstract

Despite their abundance, the molecular functions of long non-coding RNAs in mammalian nervous systems remain poorly understood. Here we show that the long non-coding RNA, NEAT1, directly modulates neuronal excitability and is associated with pathological seizure states. Specifically, NEAT1 is dynamically regulated by neuronal activity *in vitro* and *in vivo*, binds epilepsy-associated potassium channel-interacting proteins including KCNAB2 and KCNIP1, and induces a neuronal hyper-potentiation phenotype in iPSC-derived human cortical neurons following antisense oligonucleotide knockdown. Next generation sequencing reveals a strong association of NEAT1 with increased ion channel gene expression upon activation of iPSC-derived neurons following NEAT1 knockdown. Furthermore, we show that while NEAT1 is acutely down-regulated in response to neuronal activity, repeated stimulation results in NEAT1 becoming chronically unresponsive in independent *in vivo* rat model systems relevant to temporal lobe epilepsy. We extended previous studies showing increased NEAT1 expression in resected cortical tissue from high spiking regions of patients suffering from intractable seizures. Our results indicate a role for NEAT1 in modulating human neuronal activity and suggest a novel mechanistic link between an activity-dependent long non-coding RNA and epilepsy.

Long non-coding RNAs (lncRNAs) have appeared relatively recently in evolution^1^ and have significantly expanded, especially in the human genome. Strikingly, around one-third of lncRNAs are primate-specific^2^ and display specific spatial and temporal expression^3^,^4^. Emerging evidence is accumulating that demonstrates important roles for lncRNAs in regulating brain development and function^5^, as well as diseases such as schizophrenia^6^, when dysregulated. They often contain multiple functional domains that allow binding to RNA, DNA and protein in scaffolds^7^, which enables coordinate organization of regulatory circuits with diverse functions.

The lncRNA Nuclear-Enriched Autosomal Transcript 1 (NEAT1) is essential for formation of enigmatic subnuclear domains called paraspeckles, which are found in all mammalian cells^8^,^9^. Although the function of paraspeckles remains poorly understood, they have been shown to dynamically promote the retention of specific edited mRNA transcripts within the nucleus during differentiation *in vitro* to control developmental timing^8^,^10^,^11^,^12^. Immune stress^13^ and proteasome inhibition^14^ have also been demonstrated to be potential regulators of paraspeckle function. In addition to promoting paraspeckle formation, NEAT1 binds proteins such as amyotrophic lateral sclerosis (ALS)-associated proteins TDP-43, FUS/TLS^9^, and particular chromatin loci^15^. These results have led to the view that NEAT1 acts as a nuclear scaffold that is functionally responsive to specific cellular triggers. Notably, although NEAT1 is essential for nuclear paraspeckle formation, NEAT1 knockout mice show no obvious developmental phenotype^12^, suggesting a more subtle regulatory role. It is important to note, however, that no comprehensive cognitive testing has yet been reported for NEAT1 knockout mice.

Here, we show that NEAT1 is transiently down-regulated in neurons in an activity-dependent fashion and binds potassium channel-interacting proteins that are themselves strongly linked to seizure activity upon dysregulation. We further find that NEAT1 directly regulates neuronal excitability in human induced pluripotent stem cell (iPSC)-derived neurons and in next generation sequencing studies where NEAT1 was down-regulated by antisense oligonucleotides (ASOs) in iPSC-derived neurons. Using human epilepsy patient samples and *in vivo* rat seizure models, we show that excessive excitability results in chronic insensitivity of NEAT1 to neuronal activation. These data, combined with published associations between NEAT1, KCNAB2, KCNIP1 and seizure risk, establish a novel lncRNA-mediated molecular mechanism in human neurons linked to epilepsy.

## Results

### NEAT1 binds potassium channel-interacting proteins important for modulating neuronal excitability

**Figure 1:**
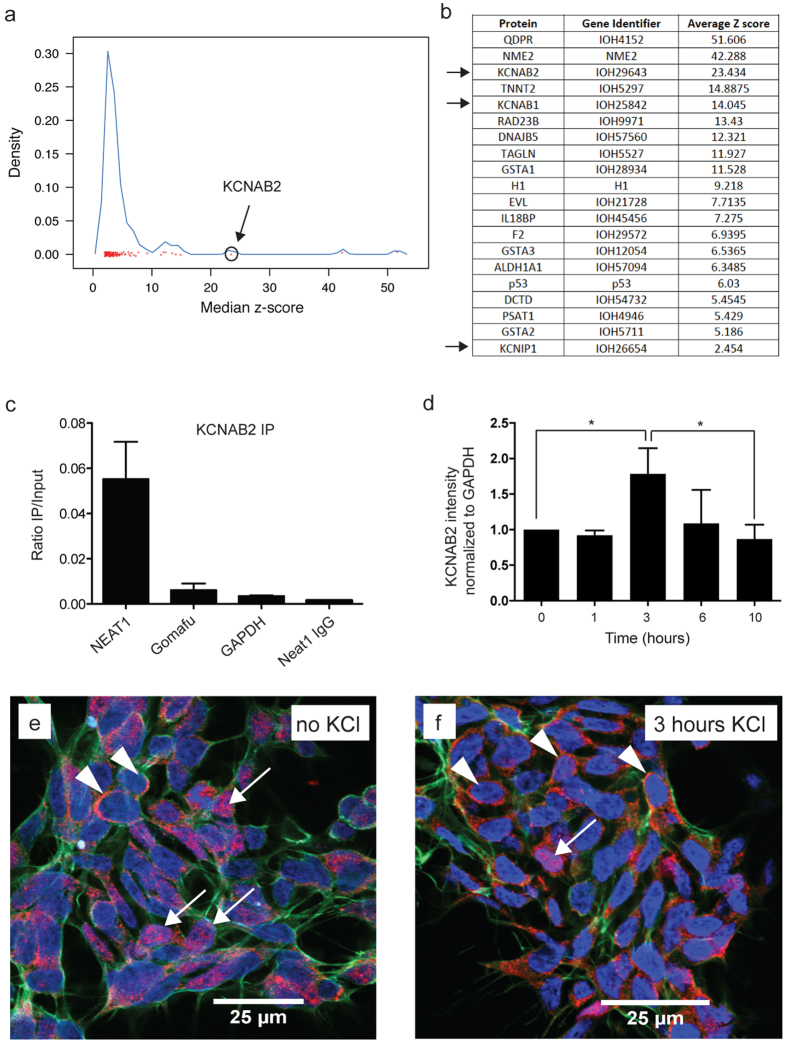
NEAT1 binds the potassium channel-interacting protein KCNAB2 that is enriched in the cytoplasm upon neuronal activation. (**a**) Protein microarray reveal potential NEAT1 interacting proteins with KCNAB2 circled as a high confidence hit. (**b**) Table representing the highest confidence hits includes KCNAB2 together with other potassium channel-interacting proteins KCNAB1 and KCNIP1 (arrows). (**c**) KCNAB2 binds directly to NEAT1 as determined via RNA immunoprecipitation in the neuroblastoma cell-line SH-SY5Y. (**d**) Activation of SH-SY5Y cells with 50 mM KCl results in a significant increase of KCNAB2 protein in the cytoplasm after 3 hours using western blot analysis with a return to baseline after 10 hours. (n ≥ 3, *p value < 0.05; One-way ANOVA test with a Tukey’s multiple comparison post hoc test) (**e**) Immunohistochemistry in SH-SY5Y cells shows that KCNAB2 (red) is mostly nuclear localized (see arrows) with few cells showing KCNAB2 cytoplasmic staining (see arrowheads). (**f**) In contrast, 3 hours post KCl activation, KCNAB2 protein is mostly cytoplasmic (see arrowheads).

To gain further insight into the underlying mechanism of NEAT1 function we initially interrogated a human proteome microarray^16^ consisting of over 16,000 different full-length human proteins (Supplementary Table 1). This analysis identified, among others, members of the potassium channel-interacting family, including KCNAB2 (Fig. 1a,b), as high confidence protein binding partners for NEAT1. These proteins are known to reduce neuronal excitability in order to protect neurons from excessive activity^17^,^18^ and were previously implicated in epileptic seizure activity^19^,^20^. After validating that KCNAB2 (Fig. 1c) and KCNIP1 (Supplementary Fig. 1) proteins indeed directly bound NEAT1 in the neuroblastoma cell-line SH-SY5Y by native RNA immunoprecipitation, we found that activation of SH-SY5Y cells by depolarization with 50 mM KCl results in a significant and transient cytoplasmic enrichment of KCNAB2 (Fig. 1d) with a corresponding significant nuclear decrease (Supplementary Fig. 2a) and a significant reduction in NEAT1 transcript after 1 and 3 hours (Supplementary Fig. 2b). We validated that the SH-SY5Y cells were activated following KCl administration via the expected increase in transcript levels of immediate early genes (IEGs) FOS and ARC (Supplementary Fig. 3). Furthermore, we show by immunohistochemistry that KCNAB2 protein is mostly nuclear in inactivated cells (Fig. 1e) but becomes mostly cytoplasmic after 3 hours post KCl activation (Fig. 1f) supporting our western blot analysis data.

### NEAT1 expression level is activity-dependent and directly modulates neuronal excitability in iPSC-derived neurons

**Figure 2:**
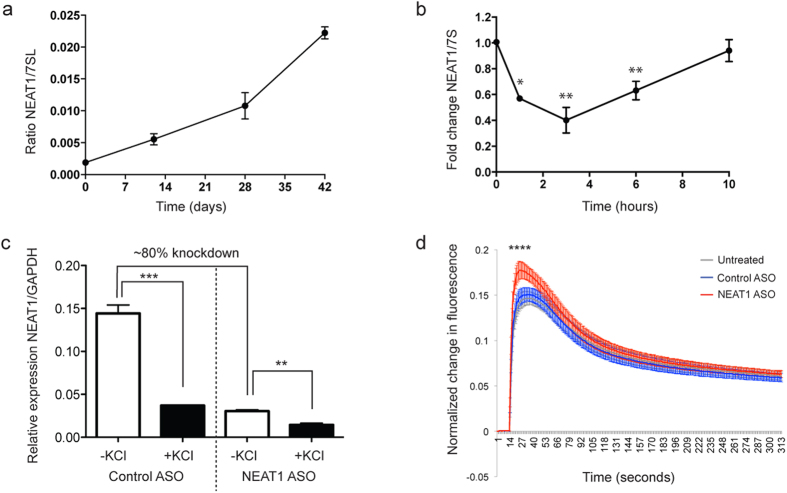
NEAT1 is down-regulated following neuronal activity and modulates neuronal excitability. (**a**) NEAT1 expression increases during cortical-type neuronal differentiation. (**b**) NEAT1 is acutely and transiently down-regulated following depolarization in human induced pluripotent stem cell (iPSC)-derived neurons (n ≥ 3; One-way ANOVA test with a Tukey’s multiple comparison post hoc test; *p value < 0.05; **p value < 0.01). (**c**) Antisense oligonucleotides (ASOs) directed at NEAT1 successfully reduced NEAT1 transcript by around 80% and knockdown did not affect the ability of neuronal activation to significantly reduce residual NEAT1 levels. (n = 3; unpaired Student’s t-test; **p value < 0.01; ***p value < 0.001). (**d**) Antisense oligonucleotide (ASO)-mediated down-regulation of NEAT1 results in enhanced depolarization-induced calcium influx in iPSC-derived neurons. (n ≥ 300 independent wells; One-way ANOVA test with a Tukey’s multiple comparison post hoc test; ****p value < 0.0001).

To investigate the link between NEAT1, potassium channel proteins, and seizure risk in a relevant human cellular system, we generated functional human cortical-type neurons from human induced pluripotent stem cells (iPSCs) [Supplementary Fig. 4a–e^6^]. Robust cortical differentiation^21^ was verified using a panel of neuronal markers, showing that the *in vitro* neuronal differentiation protocol recapitulated major *in vivo* cortical differentiation stages (Supplementary Fig. 5a–c). MAP2 immunoreactivty revealed extensive neural fiber networks present in mature cultures (Supplementary Fig. 5d,e). We found that NEAT1 expression is up-regulated during iPSC-derived neuronal differentiation (Fig. 2a) and is localized to the nucleus in mature iPSC-derived neurons, as expected^8^, using fluorescence *in situ* hybridization (Supplementary Fig. 5f).

Interestingly, KCl-induced depolarization of cortical iPSC-derived neuronal cultures resulted in a transient down-regulation of NEAT1 (Fig. 2b), with temporal dynamics that closely mirrored the activity-dependent cytoplasmic increase in KCNAB2 protein (Fig. 1d). We verified that KCNAB2 localization was also altered in iPSC-derived neurons upon KCl-induced activation (Supplementary Fig. 6) as was observed in SH-SY5Y cells (Fig. 1e,f). To investigate the functional significance of activity-dependent NEAT1 down-regulation, we knocked down NEAT1 transcript using ASOs directed against NEAT1 (Fig. 2c) and measured the effect of this on neuronal excitability in iPSC-derived neurons through Fluorescent Imaging Plate Reader (FLIPR) intracellular calcium mobilization assays, as intracellular calcium changes are critical for synapse-induced neuronal responses^22^. Notably, ASO-mediated NEAT1-knockdown significantly increased depolarization-induced calcium influx (Fig. 2d), demonstrating that NEAT1 may modulate neuronal excitability.

### Next generation sequencing of iPSC-derived neurons following NEAT1 knockdown implicates NEAT1-dependent ion channel modulation

To investigate genome-wide transcriptomic changes induced by NEAT1 knockdown, we performed whole transcriptome RNA sequencing (RNAseq) on iPSC-derived neurons, with and without KCl-induced depolarization, and before and following ASO-mediated NEAT1 knockdown. We found extensive and significant transcript changes between KCl-treated and mock-depolarized samples, with and without NEAT1 ASOs (Supplementary Fig. 7a), including the expected activation of IEGs such as FOSB, JUNB and PLK2 (Supplementary Fig. 7b and qRT-PCR validation shown in Supplementary Fig. 8). It is notable that the levels of activation of these IEGs was stronger in NEAT1-ASO-treated cells relative to controls, in agreement with our data showing increased neuronal activation after NEAT1 depletion (Fig. 2d). Since only modest expression differences were identified between inactivated NEAT1-knockdown and control neurons (Supplementary Fig. 7a), these regulatory functions of NEAT1 appear restricted to activity-dependent contexts.

**Figure 3:**
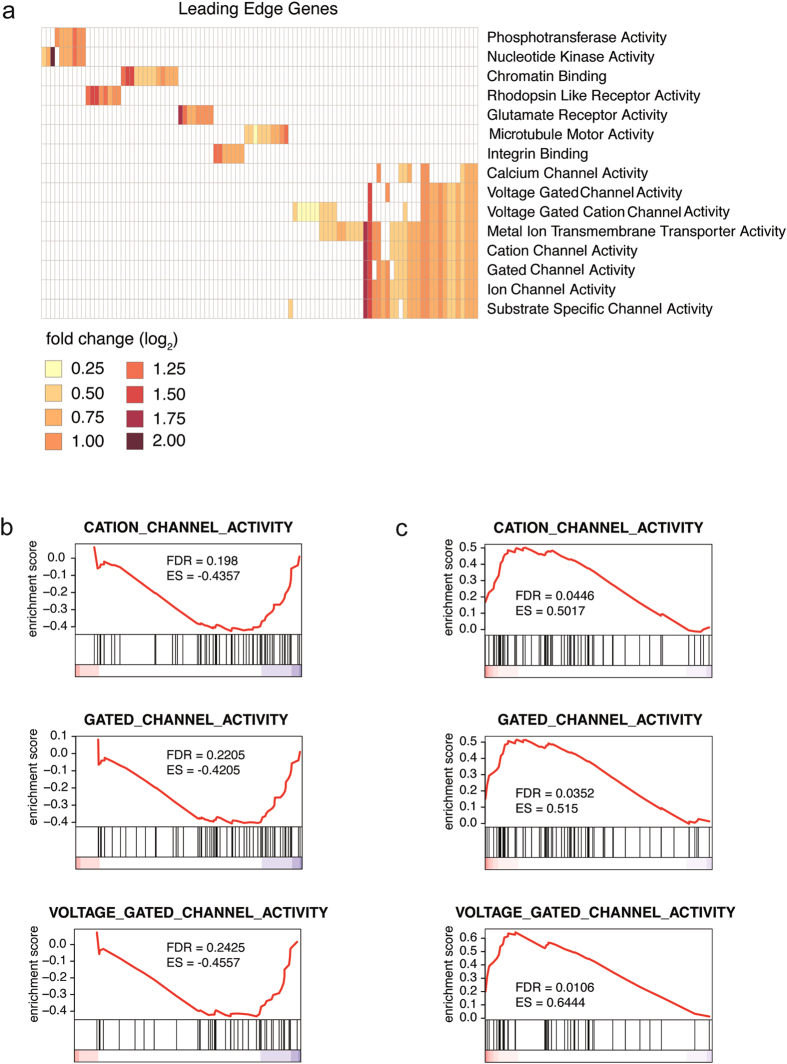
NEAT1 is associated with ion channel function. (**a**) Deep sequencing was performed on iPSC-derived neurons that were subjected to ASO-mediated NEAT1 knockdown followed by KCl-induced activation. Leading edge gene analysis revealed a strong correlation between NEAT1 expression and significantly altered genes with ion channel classifications. Specific genes included in significant gene sets are shown in Supplementary Table 3. (**b**) Gene set enrichment analysis (GSEA) of activated control ASO-treated iPSC-derived neurons demonstrated that endogenous levels of NEAT1 transcript are inversely correlated with an overall decreased expression of genes contained in ion channel gene sets relative to controls in activated neurons. The x-axis contains all the genes in the particular gene set from up-regulated (red) to down-regulated (blue). False discovery rates (FDR) and enrichment scores (ES) evaluate whether the genes in the gene set are enriched as up- or down-regulated (**c**) Conversely, ASO-mediated NEAT1-knockdown is sufficient to drive an overall elevated expression of genes contained in ion channel gene sets relative to controls in activated neurons.

We next used gene set enrichment analysis [GSEA]^23^; a method that assesses the concordance between a defined set of genes and different biological states and is, therefore, extremely useful for the unbiased identification of potential roles of transcripts with unknown function. Leading edge gene analysis, a segment of the GSEA package, was performed on genes that were significantly altered (a total of 4,623 genes) when neurons that had reduced NEAT1 levels, following treatment with NEAT1 ASOs, were activated with KCl and harvested after 3 hours (Supplementary Fig. 7a; Neat1 ASO KCl versus Control ASO KCl). The underlying premise is that sets of genes that are similarly dynamically regulated in a system share a common function. This analysis revealed that NEAT1 expression was significantly associated with expression of multiple ion channel activity gene sets (Fig. 3a). These represent the top regulated gene sets upon neuronal activation following NEAT1 knockdown. We next investigated whether the genes in these enriched gene sets were significantly up or down-regulated in our experimental conditions. First, for the control condition, GSEA performed on 5,339 significantly altered genes from mock-treated control iPSC-derived neurons following activation (Supplementary Fig. 7a; Control ASO KCl versus Control ASO) showed that NEAT1 expression is correlated with decreased ion channel gene set expression (Fig. 3b) implying a normal role in decreasing ion channel excitation. Second, to demonstrate that this correlation arises from a direct role of NEAT1 in regulating ion channel gene expression, we showed that NEAT1 knockdown in activated neurons (Supplementary Fig. 7a; Neat1 ASO KCl versus Control ASO KCl) is itself sufficient to drive a significant increase in ion channel gene set expression relative to controls (Fig. 3c) demonstrating that NEAT1 is necessary for the proper modulation of neuronal excitability. Gene ontology (GO) analysis of genes differentially expressed in activated NEAT1-knockdown neurons relative to activated controls further revealed an enrichment for apoptosis, cell death and stress related pathways, suggesting that reduction of NEAT1 may induce a neuronal stress response (Supplementary Figs 9 and 10).

### Dynamic and activity-dependent dysregulation of NEAT1 expression is associated with seizure states *in vivo*

**Figure 4:**
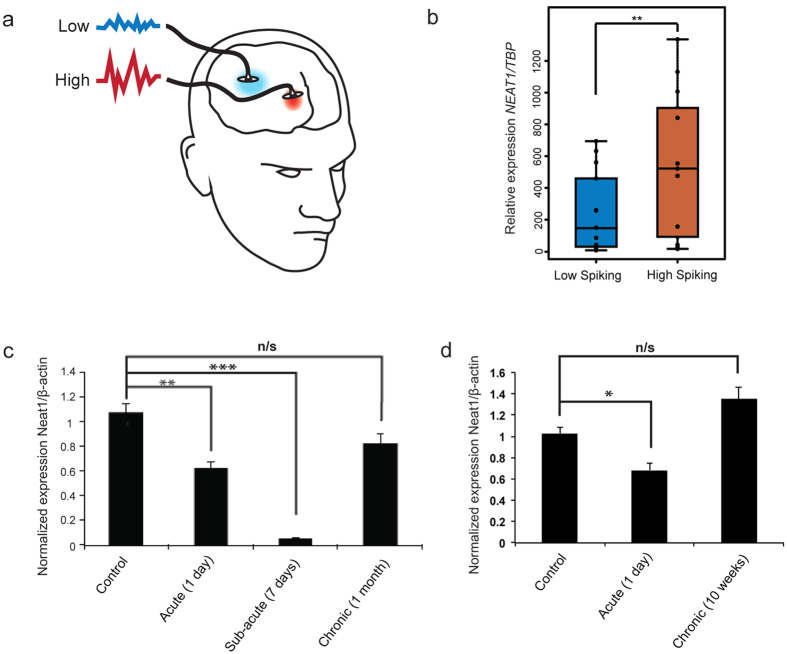
NEAT1 expression is up-regulated in human epilepsy samples and in chronically stimulated *in vivo* rat models. (**a**) Regions were resected that displayed aberrant high or low activity in the cerebral cortex of epilepsy patients. (**b**) Quantitative PCR performed on resected human epilepsy patient tissue samples showed a significant increase in NEAT1 transcript levels between high and low activity regions (n = 14, Student’s t-test; **p value < 0.01). (**c**) NEAT1 is acutely down-regulated but chronically insensitive in an *in vivo* rat model of pilocarpine-induced seizures (n ≥ 3, **p value < 0.01, ***p value < 0.001; One-way ANOVA test with a Tukey’s multiple comparison post hoc test). (**d**) Similarly, NEAT1 is acutely down-regulated but chronically insensitive in an *in vivo post-*KA induced Status Epilepticus (SE) model. (n = 3, *p value < 0.05; One-way ANOVA test with a Tukey’s multiple comparison post hoc test).

To understand potential disease-related functions of NEAT1, we examined existing literature, and found that the chromosomal locus containing NEAT1, 11q13, is in fact a region implicated in idiopathic generalized seizures^24^. Furthermore, NEAT1 was previously found to be up-regulated in a small cohort of patients with intractable seizures^25^. We then extended these initial observations with an additional 14 seizure patient samples, and found that these also display significantly up-regulated NEAT1 expression in high versus low activity regions resected from the brains of affected individuals (Fig. 4a,b).

These convergent ties between NEAT1 and epilepsy led us to speculate that our results may thus provide a mechanistic basis for these associations and explain how NEAT1 could drive excessive cortical neuronal activity characteristic of epilepsy^26^. However, since we found that knockdown of NEAT1 increased neuronal excitability (Fig. 2b), it was initially unclear why NEAT1 should be up-regulated in seizure patient brains. To explain this apparent discrepancy, we hypothesized that NEAT1 expression changes may vary according to temporal stimulation. To investigate this hypothesis, we employed *in vivo* rat models of chronic seizures. First, we found that acute (1 and 7 days) pilocarpine-induced seizure activity, a well-established model of temporal lobe epilepsy [TLE^27^], significantly down-regulated NEAT1 expression (Fig. 4c), consistent with our findings in iPSC-derived neurons where NEAT1 was also down-regulated upon acute stimulation. However, after one month, seizure-dependent NEAT1 down-regulation in response to chronic pilocarpine-induced seizure activity interestingly disappeared (Fig. 4c), perhaps due to homeostatic feedback. Second, we employed the hippocampal-specific kainic acid-induced seizure rat model that also closely resembles features of TLE^28^. Here, we again saw an acute (24 hours) down-regulation of NEAT1 and a chronic (10 weeks) insensitivity to seizure activity (Fig. 4d). These results, therefore, unify our findings (i.e. acute, activity-dependent down-regulation of NEAT1) with that of the literature (i.e. loss of NEAT1 down-regulation in response to chronic cellular stressors^11^,^13^). This suggests that in neurons, NEAT1 expression is down-regulated in response to acute stimulation but develops insensitivity upon chronic stimulation leading to an increased risk of seizure activity.

## Discussion

Epilepsy is characterized by excessive cortical neuron activity^26^, with multiple molecular pathways involved. For example, previous studies have implicated disruptions of synaptic modulatory pathways^29^ and alterations in ion channel activity^30^. However, how these pathways converge to contribute to the disease and whether they are cause or consequence remains largely unknown. Nuclear lncRNAs are emerging as common structures for providing activity-dependent platforms for the sophistication of functional processes in higher organisms by bringing together many cellular components into discrete functional entities. Here we provide evidence that the lncRNA NEAT1, previously suggested to be involved in human intractable seizure tissue^25^, contributes to the modulatory capacity of neuronal excitability and that dysregulation thereof renders neurons susceptible to seizure activity. We show that expression of NEAT1 is down-regulated in response to acute neuronal activity and binds ion channel modulatory proteins such as the potassium channel-interacting protein KCNAB2, which is itself associated with neuronal excitability^17^ and epilepsy^19^. We interpret these data to mean that NEAT1 may provide a scaffolding function in the nucleus that upon neuronal activation, releases modulatory proteins to fine-tune the excitatory response. Furthermore, our findings also show that downregulation of NEAT1 may result in changes to the expression of multiple gene transcripts involved in ion channel function following neuronal activation. These data point to NEAT1-associated paraspeckles either directly (e.g. through the binding of pre-mRNAs or guiding mRNA modifications on these transcripts) or indirectly (e.g. through sequestering transcript modifying proteins or upstream transcription factors) exerting a broad effect on downstream ion channel associated function.

NEAT1 is widely expressed in the human body and other studies have also reported an up-regulation of NEAT1 in response to other forms of chronic stress, for example, in the placenta of patients with Intrauterine Growth Restriction (IUGR)^31^, and in response to stressors such as proteasome degradation^14^ and viral infection^13^,^32^. Therefore, it may be that NEAT1 regulates a common mechanism of activity-dependence and chronic stress-induced insensitivity whose functional specifics are contingent on cell-type. Such a function is consistent with the emerging view that lncRNAs act as molecular scaffolds through their binding to many cellular components^7^. Our results thus propose a novel and therapeutically relevant lncRNA-mediated molecular mechanism in human neurons. Future studies would be required to explore the ways in which NEAT1 may function as a common regulatory hub regulating signaling pathways linked to seizure states and other normal and pathological physiological conditions.

## Methods

### Human proteome microarray

Elucidation of NEAT1 protein-binding partners was conducted using a human proteome microarray as detailed in Jeong *et al*.^16^ and analyzed using GENEPIX PRO 5.0 (Molecular Devices, CA, USA) that was used to scan the chip images. For every spot, signal intensity was defined as foreground median intensity divided by its local background median intensity. Two rounds of normalization were applied. First, within-chip normalization was used to adjust the median signal intensity of each block to 1. Second, whole chip normalization was applied to standardize this set of signal intensities to a mean of 0 and standard deviation of 1, with the resulting normalized signal intensity called the z-score. Arrays contained two identical probes corresponding to each protein and were repeated three times. To be considered as a positive interaction, probes corresponding to a protein were required to have an average z-score of ≥2 (P < 0.0015) over the array experiments conducted, which resulted in 92 positive hits. Median z-score for the six conducted replicates is presented in Fig. 1a as a kernel density plot. NEAT1 probes were synthesized as follows: Probes: Neat1-1 forward primer: 5′GCTGGAGTCTTGGGCACGGC, Neat 1-1 reverse primer 5′TCAACCGAGGCCGCTGTCTC; Neat1-2 forward primer 5′AAATTGTTTTGCTTCACTGT, Neat1-2 reverse primer 5′GATAGCAGCATTGGAATTCT. Briefly, two single-stranded complementary RNA (cRNA) probes were synthesized from genomic DNA. These two probes were assayed on the human protein microarray to determine the protein hits specific to NEAT1. A graphical representation of median z-scores (Supplementary Table 1) for significant protein hits is shown in Fig. 1a.

### RNA immunoprecipitation and western blot analysis

#### RNA immunoprecipitation

RNA immunoprecipitation was performed as previously described^6^. At least 3 independent experiments were performed to investigate potential interactions. KCNAB2 and KCNIP1 antibodies were purchased from LifeSpan Biosciences Inc. (WA, USA).

#### Cell fractionation

SH-SY5Y cell fractionation was performed according to the protocol described by Suzuki *et al*.^33^.

#### Western blot analysis

Antibodies used were mouse anti-TBP (ab818, Abcam, UK), mouse anti-GAPDH (ab8245, Abcam, UK) and rabbit anti-KCNAB2 (LSBio; LS-C30846, USA). Protein signals were detected and quantified by SuperSignal West Pico Chemiluminescence Substrate (Thermo Scientific, USA) on the Vilber Fusion-FX Chemiluminescence System (Vilber Lourmat, USA).

### Induced pluripotent stem cell culture conditions

Human iPSC-derived neurons were generated from iPSC bulk cultures as previously described^6^. Our differentiation and activation protocol matched the protocol recently described by Hook *et al*.^34^, where they showed robust KCl-induced neurotransmitter release further strengthening the assertion that our neurons are relevant for the investigation into endogenous human neuron function. IPS cell lines used were H9, C11 and C32. C11 iPSCs were derived from male fibroblasts (ATCC CRL2429) and C32 iPSCs from female fibroblasts (ATCC CRL1502). All iPSC experiments used 3 technical replicates from each of these 3 cell lines. Primer sequences for quantitative PCR analysis are listed in Supplementary Table 2 and statistics were determined using an unpaired t-test (n > 3).

### Fluorescence *in situ* hybridization (FISH)

Stellaris RNA FISH probes (Biosearch Technologies, CA, USA) were used, with 48 × 20 mer fluorophore conjugated oligos tiling the length of the target transcript. GAPDH and NEAT1 probe sets were supplied as predesigned controls conjugated to Quasar 570 fluorophores. Staining was carried out as described in the Stellaris protocol for adherent mammalian cells, but adapted for cells grown on coverslips using 15% rather than 10% formamide for hybridization, which was found to reduce background signal. GAPDH and NEAT1 probe sets were used at 50 nM. IPSC-derived matured neurons were imaged using a Zeiss LSM 710 confocal microscope (Zeiss, Oberkochen, Germany) with manufacturer provided ZEN imaging software. Presented images are maximum intensity projections of 20 Z-stack slices taken with a standard 1 AU slice depth and 0.5 AU intervals, capturing the entire volume of the cell. Other than linear contrast/brightness adjustments the images are unmodified. Unstained cells used as a negative control showed no fluorescence signal using identical imaging settings.

### Immunohistochemistry

#### SH-SY5Y cells

Coverslips were placed in a 24-well plate, coated with Poly-L-Lysine for 5 minutes and dried overnight after washing four times with water. SH-SY5Y cells were plated at a density of 2.5 × 10^5^ in 500 ul of DMEM with 10% FBS and grown overnight. The cells were fixed in 4% paraformaldehyde/4% sucrose in phosphate-buffered saline (PBS) at room temperature for 15 minutes and washed three times for 5 minutes in PBS. *iPSC-derived neurons:* Approximately 15,000 neurons were seeded into each well of an 8-well chamber slide (Thermo Fisher), previously coated in Matrigel (BD). Neurons received either mock treatment or application of 50 mM KCl in N2B27 neurobasal media. Depolarized cells were fixed at one, three and six, hours post application of KCl. Cells were washed once gently, so as not to disturb fragile cell extensions, in PBS and fixed in 4% paraformaldehyde, 4% sucrose for 15 minutes at room temperature. *Immunohistochemistry:* Cells were then permeabilized in 0.1% Triton X-100/0.1% Na-Citrate/PBS for 3 minutes at room temperature and washed three times for 5 minutes with PBS. Cells were next blocked in 10% FBS/PBS for 1 hour at room temperature and incubated with primary antibodies in blocking solution overnight at 4 °C. The following day cells were washed 3 times in PBS for 5 minutes at room temperature. Then cells were incubated with secondary antibodies for 90 minutes at room temperature and washed 4 times in PBS for 5 minutes. Antibodies used were: KCNAB2 antibody (Proteintech; 1:100), MAP2 (1:1000, Millipore), Cy3 (1:500, Thermo Fisher Scientific), DAPI (1:100, Thermo Fisher Scientific), Alexa Fluor 488 Phalloidin (1:500, Thermo Fisher Scientific). Coverslips were dipped in demineralized H_2_O and mounted using MP Biomedicals immuno-fluore mounting medium. Confocal images were acquired using LSM700 or LSM710 confocal laser-scanning upright microscopes (Zeiss) with 10X, 40X (water) or 63X (oil) objectives. The zoom was set between 1× and 2× according to the cell size, with a pinhole of 34 μm and a speed of 1.58 μs per pixel. The confocal laser intensity was set to 2 and the gain was adjusted per experiment for optimal result. The dimensions were set to 512 × 512 pixels with 4 times averaging. For each condition 5 pictures were taken and all experiments were repeated 3 times. A script was written in Fiji 1.49 (ImageJ) that automatically calculated the surface area of the KCNAB2 staining, DAPI staining (nuclear surface area) and Phalliodin staining (cytoplasmic surface area). The percentage of nuclear and cytoplasmic surface area that contained staining was calculated. The time points were separately compared to the control using an unpaired samples *t*-test.

### NEAT1 transcript knockdown

NEAT1 transcript knockdown was performed using antisense oligonucleotides (ASOs) developed by Isis Pharmaceuticals (Carlsbad, CA, USA) in matured human iPSC-derived neurons. These ASOs are 20-mer oligonucleotides containing a phosphorothioate backbone, 2′-*O*-methoxyethyl modifications on the first and last five nucleotides and a stretch of ten DNAs in the center. ASOs base-pair with their target RNA and cause degradation through the action of RNase H. A 10 μM final concentration of one of three different ASOs targeting NEAT1, and 10 μM of a control ASO with no predicted targets, were added to the growth media without transfection reagent. ASO-mediated knockdown of NEAT1 transcript was confirmed by qRT-PCR (Supplementary Fig. 11). As all ASOs were similar in their ability to knock down NEAT1 transcript, we have used ASO1 for the bulk of the experiments with the exception of using ASO2, in addition to ASO1, to confirm qPCR validation experiments (Supplementary Fig. 8). Statistics were determined using an unpaired Student’s t-test (n = 3). Sequences for the ASOs were as follows: Scrambled control (CCTTCCCTGAAGGTTCCTCC), NEAT1 ASO1 (ATCACACATGTAGTAAAGGC), ASO2 (TCGCTCATGATTTTCAATCA), ASO3 (ATCACACATGTAGTAAAGGC) and ASO4 (ATCATCCCCAAGTCATTGGT). Sequences for GAPDH were (Fwd: GTGAACCATGAGAAGTATGACAAC; Rev: CATGAGTCCTTCCACGATACC).

### Fluorescent Imaging Plate Reader (FLIPR)

IPSC-derived neurons were tested for calcium signaling dynamics using the FLIPR_TETRA_ High Throughput Cellular Screening System as described in Vetter *et al*.^35^. Briefly, cells were loaded with 5 μM Fluo-4-AM in physiological salt solution (PSS). To allow for completion of de-esterification, cells were incubated for 10–15 minutes with PSS. After 2 washes with PSS, cells were transferred to the FLIPR_TETRA_ (Molecular Devices, CA, USA) fluorescent plate reader and Ca^2+^ responses to activation with 50 mM KCl measured using a cooled CCD camera with excitation at 470–495 nm and emission at 515–575 nm. Camera gain and intensity were adjusted for each plate to yield a minimum of 1000 arbitrary fluorescence units (AFU) baseline fluorescence. Prior to addition of KCl, 10 baseline fluorescence readings were taken, followed by fluorescent readings every second for 300 seconds. Delta F/F was calculated for each time point as (fluorescence at time t – avg. baseline fluorescence)/(avg. baseline fluorescence) to give a normalized measure of fluorescence.

### Next generation sequencing

#### RNA preparation for library preparation

Prior to library preparation, each total RNA sample was DNase-treated using TURBO DNase (Life Technologies, CA, USA) according to the manufacturer’s instructions followed by extraction, ethanol precipitation and resuspension in nuclease-free H_2_O. DNase-treated RNA quality was assessed on an Agilent Technologies 2100 Bioanalyzer with an RNA 6000 Nano kit (Agilent Technologies, CA, USA) according to the manufacturer’s instructions. RNA concentration was measured using a Nanodrop 2000 spectrophotometer (Thermo Fisher Scientific, MA, USA).

#### Total RNA library preparation

500 ng of total RNA was used as input material for library preparation using the TruSeq Stranded Total RNA Sample Prep Kit (LT) (Illumina, CA, USA) according to manufacturer’s instructions. Individual libraries were indexed as recommended by Illumina.

#### Sequencing

For total RNA, sequencing was performed using the Illumina HiSeq2500 platform with 100 bp paired-end sequencing with a fragment size of approximately 292 bp. Illumina TruSeq version 3 chemistry was used for cluster generation and sequencing.

#### Data Processing

The paired end, stranded reads were quality filtered with Trimgalore to avoid reads with low phred score to rule out false alignments. Reads were mapped to hg19 and Gencode v.18 as a gene model using Tophat2 with default options and first strand library type^36^,^37^. The mapped reads were counted with HTSeq to known genes from Gencode v.18 using the union model coupled with reversed strand (http://biorxiv.org/content/biorxiv/early/2014/08/19/002824.full.pdf). The gene counts were normalized to counts per million (cpm) reads and to the upper quartile using the edgeR bioconductor package^38^. This package measures the dispersion towards a trended mean and has been shown to be sensitive to outliers. Genes with less than one cpm in at least three samples were eliminated from further analysis recommended from previous methods^39^. Differential expression was measured with edgeR and the P-values were adjusted with Benjamini-Hochberg multiple testing. Genes were deemed significant if they had a FDR less than 5%.

#### Quantitative PCR

Quantitative RT-PCR (qRT-PCR) was performed using the ViiA 7 Real-Time PCR System (Life Technologies, Brisbane) and SYBR Green master mix (Qiagen, Valencia, CA). The relative mRNA level analysis was done by the ΔΔCt method. All the samples were tested in triplicate from at least 3 independent replicates. Statistical analyses were performed using a two-tailed unpaired *t-*test. Error bars represent the standard error of the mean. Human primer sequences used were PLK2 (Fwd: CTACGCCGCAAAAATTATTCCTC; Rev: TCTTTGTCCTCGAAGTAGTGGT), FOSB (Fwd: AGAGGAAGAGGAGAAGCGAA; Rev CAACTGATCTGTCTCCGCC), JUNB (Fwd: ACAAACTCCTGAAACCGAGCC; Rev CGAGCCCTGACCAGAAAAGTA).

### Gene set enrichment analysis

Genes were ranked according to fold change in NEAT1 ASO-treated cells versus control ASO-treated cells and used as input for a pre-ranked GSEA using gene sets from the KEGG and Gene Ontology Molecular Function collections. Significant gene sets were selected based on an FDR < 0.25, a cutoff commonly applied for gene set discovery. Significant channel gene sets (n = 3) were subsequently analyzed in KCl-stimulated versus unstimulated neurons. Significance of these gene sets was evaluated based on FDR < 0.05. Specific genes included in significant gene sets are shown in Supplementary Table 3. The x-axis (Fig. 3b,c) shows all genes that are expressed in the samples, ranked from positive to negative fold change (i.e. most up-regulated to most down-regulated). The color-coding along the x-axis corresponds to the value of the fold change (ranging from red, up-regulated, over white to blue, down-regulated). The vertical bars indicate the position of the genes from the respective channel gene sets in the ranked list of genes along the x-axis. Based on these positions, an enrichment score and FDR-value are calculated to evaluate whether the genes in the gene set are enriched among the up- or down-regulated genes.

### Human seizure sampling and NEAT1 expression analysis

Human tissue samples were obtained with informed consent and were approved through the Wayne State University IRB. Methods were carried out in accordance with the approved guidelines. Human neocortex samples were removed from 14 patients with refractory epilepsy. For each patient the tissues were mapped to brain regions with a known amount of interictal activity (frequency of electric activity between seizures) and separated in low and high levels as described previously^25^,^40^. Briefly, total RNA was extracted from the gray matter only, purified, and reverse transcribed using SuperScript II Reverse Transcriptase (Life Technologies, CA, USA). RT-PCR was performed using Fast SYBR Green Master Mix (Life Technologies, CA, USA) in a StepOnePlus instrument (Life Technologies, CA, USA). Statistics were determined using a paired t-test. A complete experimental description and primer sequences are provided in Supplemental Experimental Procedures.

### Rat seizure models

- (1) Pilocarpine-induced epilepsy model:

Seven week old adult male Sprague-Dawley (SD) rats (Koatech, Seoul, Korea) were used, and maintained at standard room temperature (22–24 °C) with 12 hour light and dark cycle. Rats were infused with lithium chloride (127 mg/kg, Sigma-Aldrich, MO, USA) and methylscopolamine-bromide (1 mg/kg, Sigma-Aldrich, MO, USA) via intraperitoneal route 24 h and 30 min prior to pilocarpine administration, respectively. Pilocarpine hydrochloride (30 mg/kg, i.p., Sigma-Aldrich, MO, USA), a muscarinic cholinergic receptor agonist was injected to trigger status epilepticus (SE). The seizure events were scored in pilocarpine-induced rats through repeated doses of pilocarpine hydrochloride (10 mg/kg) every 30 min according to the Racine scale^41^. Racine scale was defined as adequate measurement of seizure intensity with a 5-stage spontaneous seizure scale. Diazepam (10 mg/kg, i.p. Samjin, Seoul, Korea) was injected 60 min after the onset of seizure to reduce seizure activity. After cessation of SE, rats were given 5 ml intraperitoneal injection of 0.9% saline for hydration and treated with sufficient moistened pellets soaked in Gatorade. Control rats received lithium chloride, methylscopolamine-bromide and the same amount of 0.9% saline instead of pilocarpine. The pilocarpine-treated rats for the chronic period experiments were monitored by video recording (12 h/day for 2 days) to verify the emergence of spontaneous recurrent seizures. Acute, subacute, chronic phase in pilocarpine-treated rats started at 1, 7, and 21 days, respectively. All experiments were approved by the Institutional Animal Care and Use Committee at Seoul National University Hospital (IACUC # 13-0224). Methods were carried out in accordance with the approved guidelines. (n ≥ 3; unpaired t-test). *Quantitative Real-time PCR assay:* qRT-PCRs were conducted in triplicate using SYBR RT-PCR kit (Takara Bio Inc., Shiga, Japan) in an ABI 7500 (Life Technologies, CA, USA). The qRT-PCR reactions were carried out at 95 °C for 30 s, and 35 cycles of 95 °C for 5 s and 60 °C for 34 s. PCR primers for NEAT1 were 5′-GTTCCGTGCTTCCTCTTCTG-3′ (forward) and 5′-GTGTCCTCCGACTTTACCAG-3′ (reverse). β-actin primers (forward: 5′-AAGACCTCTATGCCAACACAGT-3′, 5′-reverse: GCTCAGTAACAGTCCGCCTA-3′) were used for RNA normalization.

- (2) Kainic acid (KA)-induced epilepsy rat model:

At 9–10 weeks of age, male Wistar rats received repeated low-dose Kainic Acid (5 mg/kg ip, followed by 2.5 mg/kg ip injections every 45 min) until SE (defined as being unresponsive to external stimuli, and experiencing convulsive seizures) was initiated^28^. Four hours after the initiation of SE animals were given an injection of diazepam (5 mg/kg ip) to cease the SE. Control animals received saline (0.9%, 2 ml/kg ip) coupled with diazepam. Animals were culled at 24 hours and 10 weeks after SE. At 8 weeks post-SE, all animals underwent 2 weeks of continuous video-EEG recording (Compumedics, VIC, Australia) to identify spontaneous seizures as previously described^42^. All SE rats had at least one seizure, verified by at least 2 reviewers. Primers and conditions for qRT-PCR are as described for the pilocarpine model (n ≥ 3; unpaired t-test). All procedures involving animals were approved by the University of Melbourne Animal Ethics Committee. Methods were carried out in accordance with the approved guidelines.

## Additional Information

**How to cite this article**: Barry, G. *et al*. The long non-coding RNA NEAT1 is responsive to neuronal activity and is associated with hyperexcitability states. *Sci. Rep.* **7**, 40127; doi: 10.1038/srep40127 (2017).

**Publisher's note:** Springer Nature remains neutral with regard to jurisdictional claims in published maps and institutional affiliations.

## Supplementary Material

Supplementary Information
