# Supplementary material for: The long non-coding RNA NEAT1 is responsive to neuronal activity and is associated with hyperexcitability states
Source: Sci Rep. 2017 Jan 5;7:40127. doi: 10.1038/srep40127 (PMC5214838; doi:10.1038/srep40127)
Supplement: Supplementary Information [file srep40127-s1.pdf]

Supplementary Information

Title

The long non-coding RNA NEAT1 is responsive to neuronal activity and is associated with hyperexcitability states

Authors

Guy Barry, James A Briggs, Do Won Hwang, Sam P Nayler, Patrick RJ Fortuna, Nicky Jonkhout, Fabien Dachet, Jesper LV Maag, Pieter Mestdagh, Erin M Singh, Lotta Avesson, Dominik C Kaczorowski, Ezgi Ozturk, Nigel C Jones, Irina Vetter, Luis Arriola-Martinez, Jianfei Hu, Gloria R Franco, Victoria M Warn, Andrew Gong, Marcel E Dinger, Frank Rigo, Leonard Lipovich, Margaret J Morris, Terence J O'Brien, Dong Soo Lee, Jeffrey A Loeb, Seth Blackshaw, John S Mattick and Ernst J Wolvetang

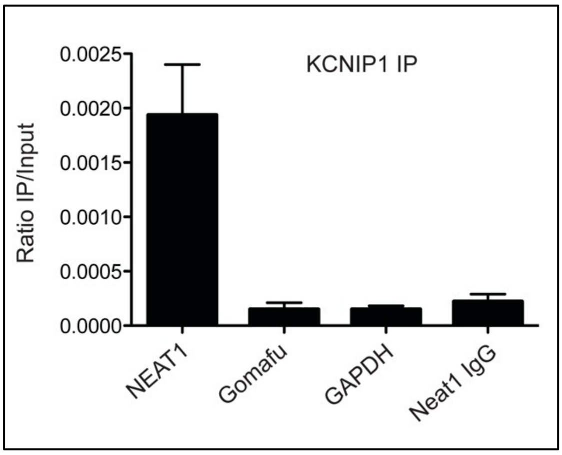

Supplementary Fig. 1: NEAT1 transcript binds directly to KCNIP1. KCNIP1 binds directly to NEAT1 transcript revealed through candidate qRT-PCR following RNA immunoprecipitation using KCNIP1 antibody (n≥3).

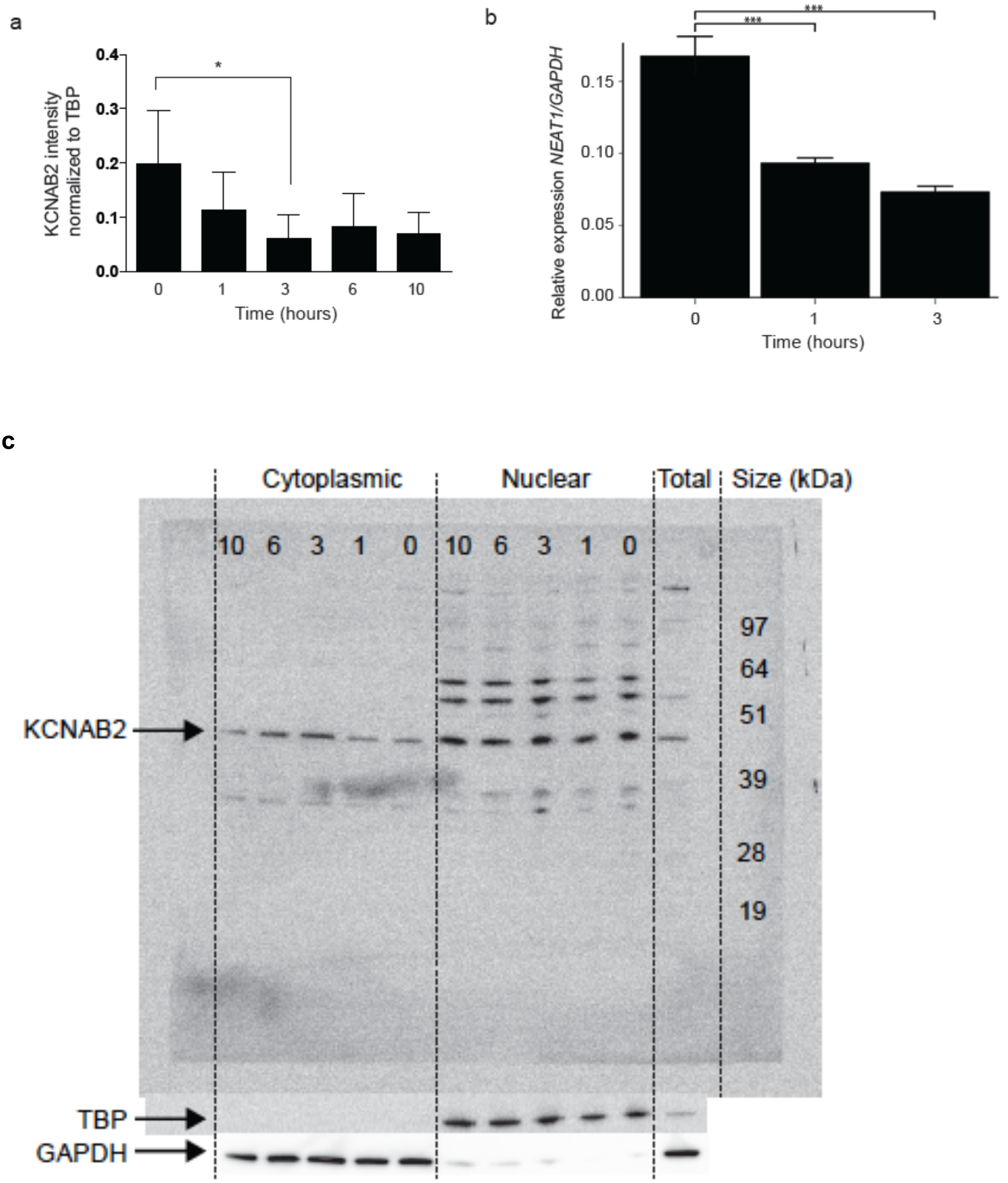

Supplementary Fig. 2: Activation of SH-SY5Y cells with 50mM KCL results in a significant decrease of KCNAB2 in the nucleus after 3 hours using western blot analysis. (a) Relative quantification (n≥3, *p value< 0.05; One-way ANOVA test with a Tukey's multiple comparison post hoc test) of KCNAB2 in the nuclear fraction, (b) qRT-PCR shows that NEAT1 transcript is significantly reduced after 1 and 3 hours post KCl activation (n=3, ***p value< 0.001; One-way ANOVA test with a Tukey's multiple comparison post hoc test) and (c) representative western blot (Nuclear-restricted TBP and cytoplasmic-restricted GAPDH were used to for purity of fractionation and relative quantification).

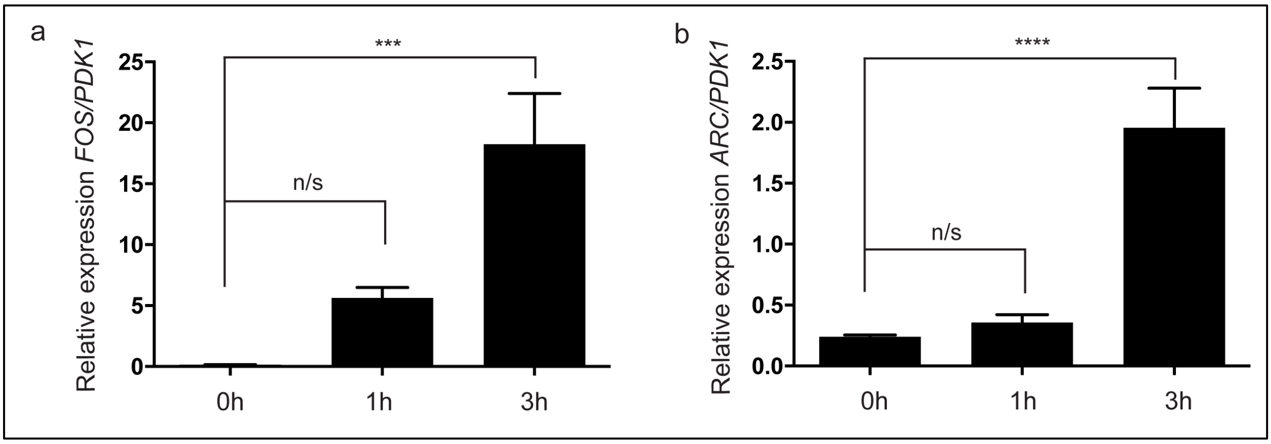

Supplementary Fig. 3: Quantitative PCR (qPCR) validation of SH-SY5Y activation. Transcript levels of the immediate early genes (a) FOS and (b) ARC were determined by qPCR and normalized to the housekeeping gene PDK1 following KCl stimulation for 1 and 3hours respectively. (n=3; One-way ANOVA test with a Dunnett's multiple comparison post hoc test ***p value< 0.001; ****p value< 0.0001).

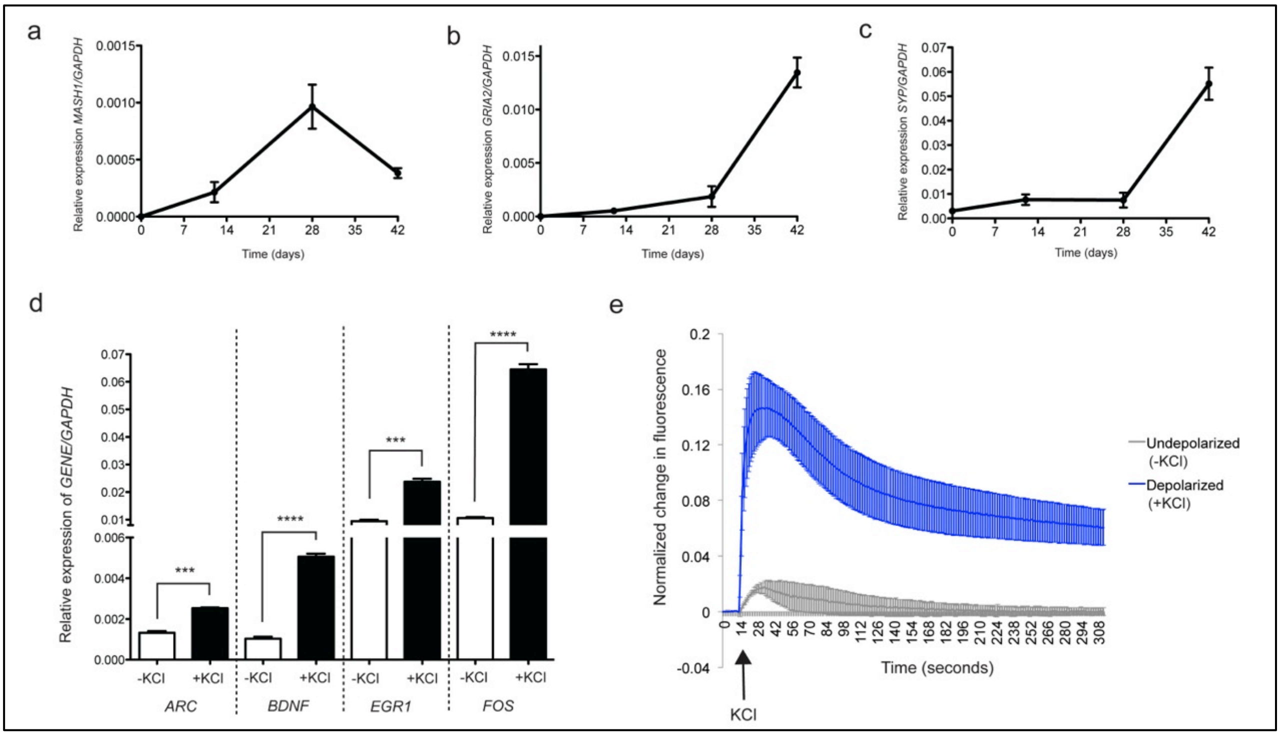

Supplementary Fig. 4: Functional neurons are generated from induced pluripotent stem cells. Markers for neuronal differentiation show that mature neurons are formed from Day 0 iPS progenitor cells. (a) The proneural gene *MASH1* was maximally expressed at day 28, while (b) the glutamate receptor subunit *GRIA2* and (c) synaptic marker *synapsin1* expression peaked during final maturation steps as expected. (d) 3 hour KCl-mediated activation of matured iPSC-derived neurons demonstrated the expected upregulation of immediate early gene transcripts such as *ARC*, *BDNF*, *EGR1* and *FOS*; (n≥3; Student's unpaired t-test; ***p value< 0.001; ****p value< 0.0001). (e) KCl-mediated activation of matured iPSC-derived neurons also demonstrated the expected increase in intracellular calcium levels using the Fluorescent Imaging Plate Reader assay.

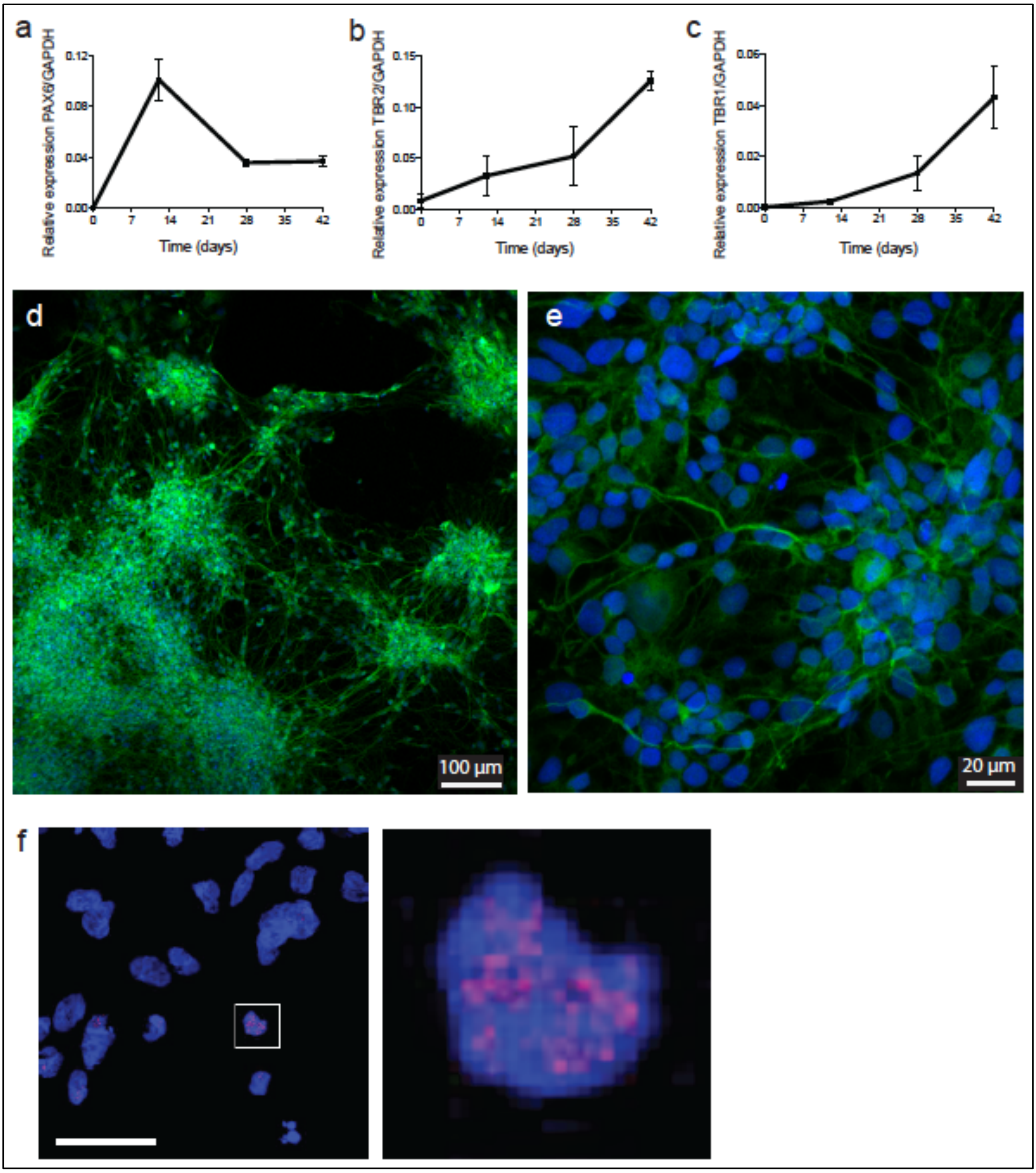

Supplementary Fig. 5: Cortical-type neurons are generated through the neuronal differentiation protocol. (a) Cortical differentiation markers for cortical progenitor cells (PAX6), (b) intermediate progenitor cells (TBR2) and (c) post-mitotic cortical neurons (TBR1) demonstrate the enrichment of the differentiation protocol for cortical-type neurons. (d, e) MAP2 antibody staining reveals extensive fiber networks present in mature iPSC-derived neuronal cultures here shown at low (10X) and higher (40X) magnification. Scale bars are as represented in the individual figures. (f) NEAT1 is localized to the nucleus (DAPI stained) of matured iPSC-derived neurons. Images are maximum intensity projections of z-stacked images capturing the entire cell. Scale bar: 30µM.

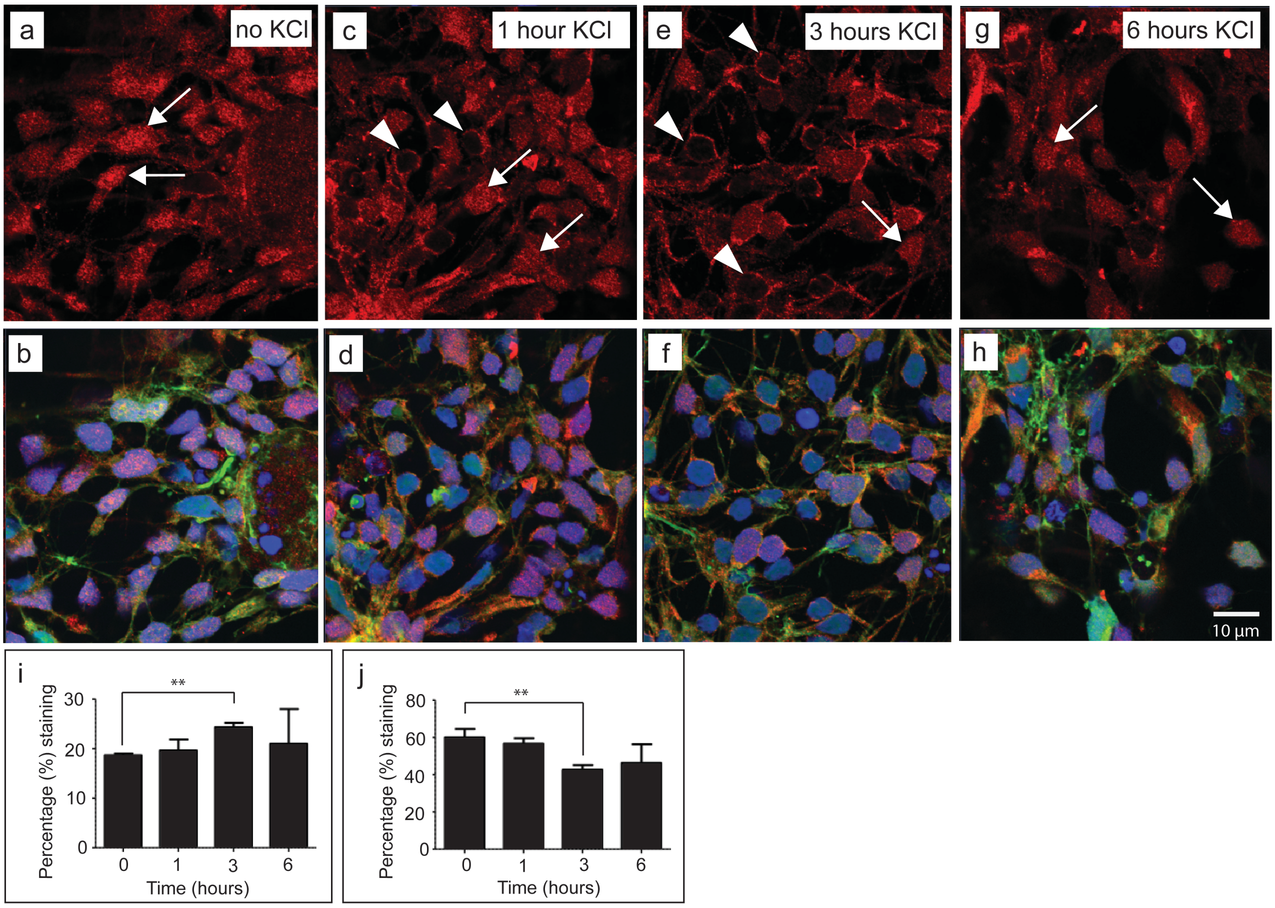

Supplementary Fig. 6: KCNAB2 localization in iPSC-derived neurons flowing KCl-induced activation. Top panels (a, c, e, g) show KCNAB2 antibody staining only while bottom panels (b, d, f, h) are inclusive of KCNAB2, DAPI and phalloidin staining. (a, b) KCNAB2 localization in untreated iPSC-derived neurons is predominantly nuclear (see white arrows for examples). (c, d) After 1 hour post activation with 50mM KCl neurons are observed with ring-like cytoplasmic-only patterns (see white arrowheads for examples). (e, f) This cytoplasmic KCNAB2 pattern is most clearly evident after 3 hours post KCl-activation. (g, h) KCNAB2 is again predominantly nuclear after 6 hours post KCl-activation. Quantification (as a percentage of total KCNAB2 staining in the cell) of cytoplasmic (i) and nuclear (j) KCNAB2 protein. KCNAB2 Red (Cy3; KCNAB2), green (Phalloidin GFP), blue (DAPI). Scale bar: 10µm

a

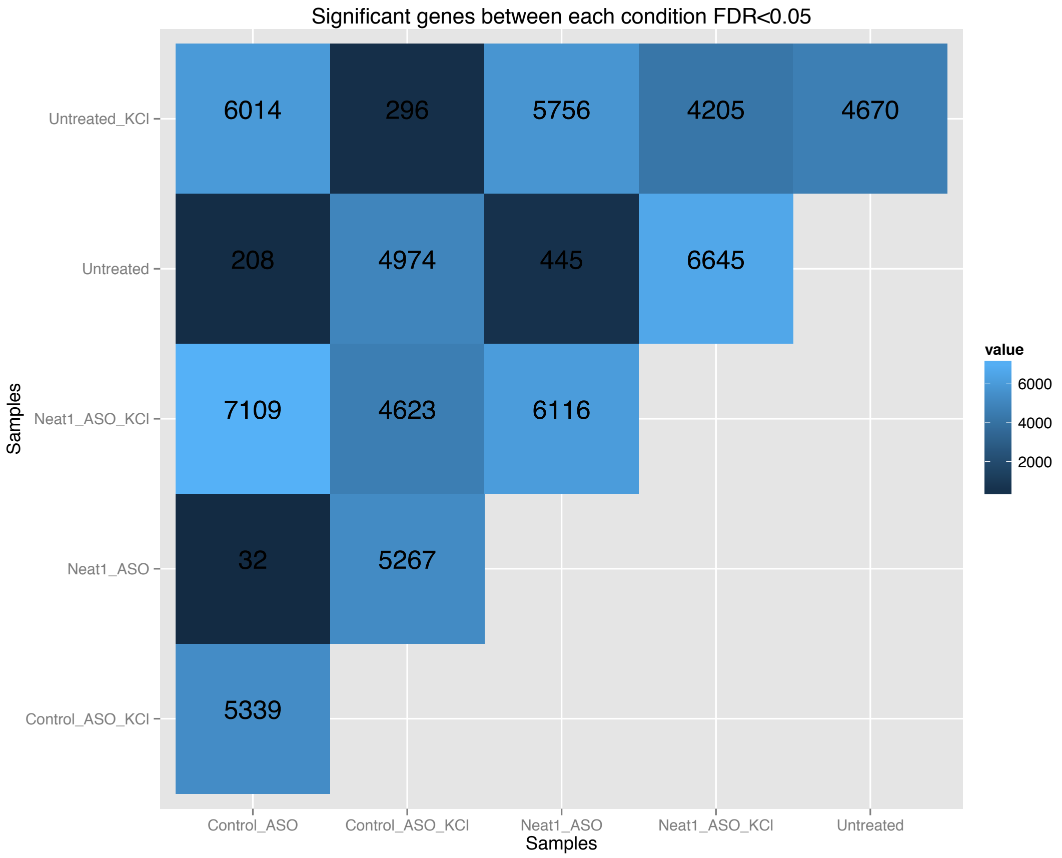

b

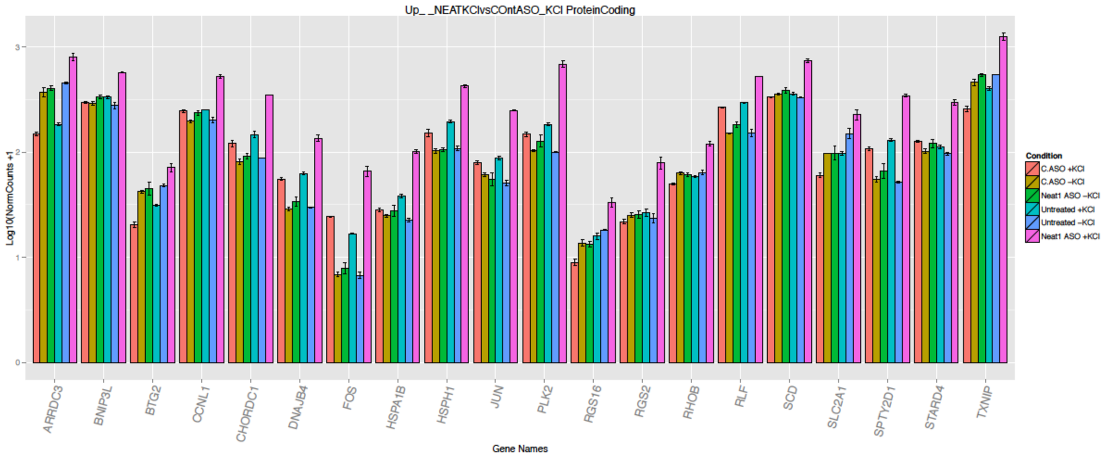

Supplementary Fig. 7: Next generation sequencing analysis using iPSC-derived neurons. (a) Numbers of genes significantly altered, either increased or decreased, in our next generation sequencing experiments. (b) Highest increases in gene expression with specific attention paid to the activated NEAT1 ASO (NEAT1 ASO +KCl) column. Expected increases in immediate early genes, such as FOS and JUN, are evident when comparing the red bars (Control ASO +KCl) with yellow bars (Control ASO –KCl). These increases are amplified following activation when NEAT1 is knocked down using NEAT-directed ASOs (pink bars; NEAT1 ASO +KCl) compared with controls (dark green bars; NEAT1 ASO -KCl).

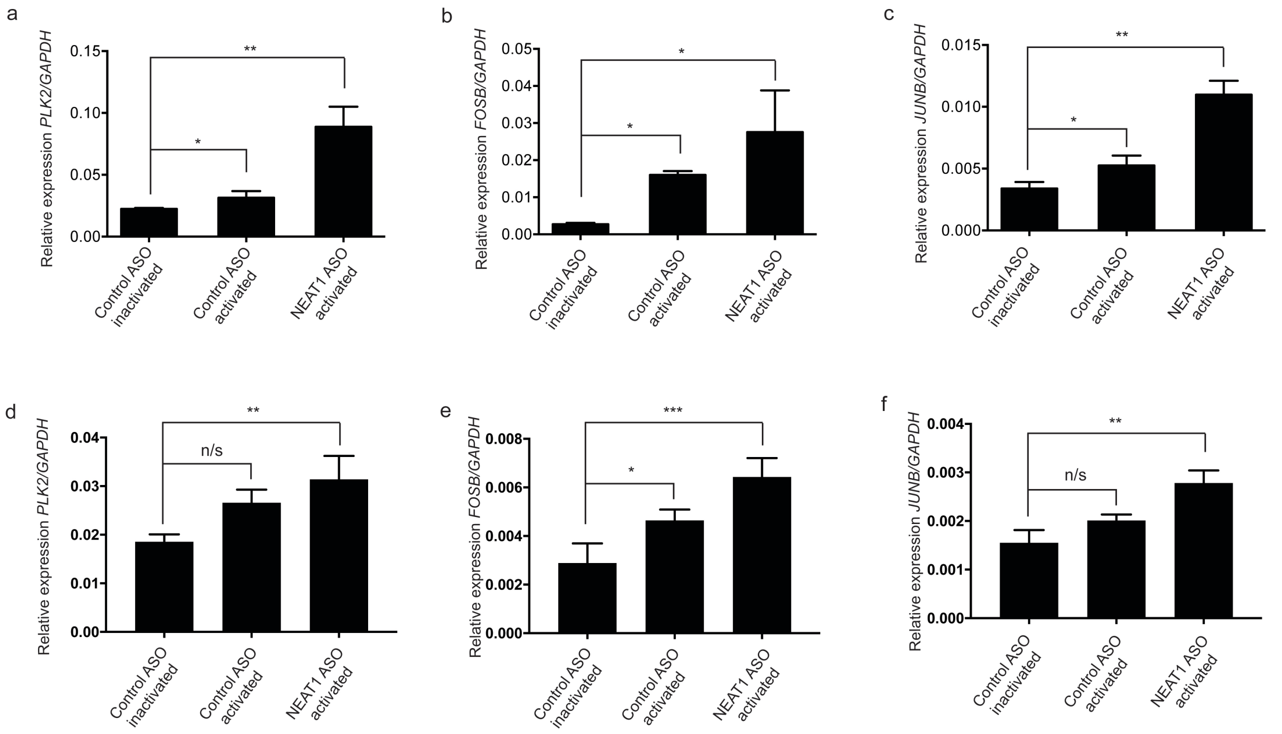

Supplementary Fig. 8: Quantitative PCR (qPCR) validation of next generation sequencing (NGS) results. Transcript levels of the immediate early genes PLK2 (a, d), FOSB (b, e) and JUNB (c, f) were determined by qPCR and normalized to the housekeeping gene GAPDH following 3 hours of KCl-induced activation, and supported the results obtained through next generation sequencing analysis. Results were obtained using 2 different ASOs targeting NEAT1, namely ASO1 (a, b, c) and ASO2 (d, e, f). (n=3; One-way ANOVA test with a Tukey's multiple comparison post hoc test *p value< 0.05; **p value< 0.01).

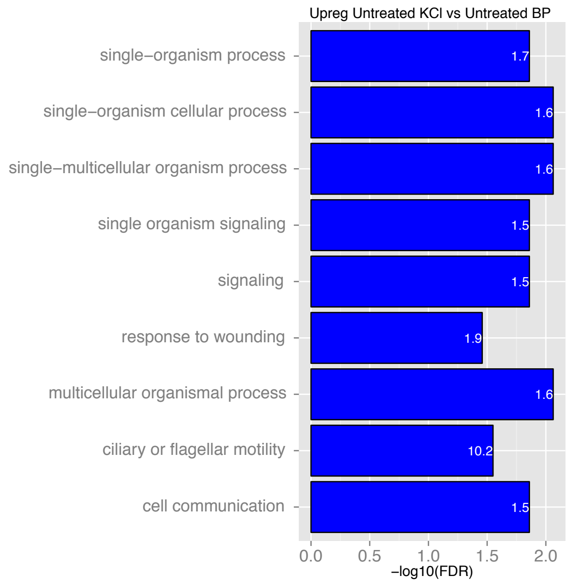
Upreg Untreated KCl vs Untreated BP
single−organism process
single−organism cellular process
single−multicellular organism process
single organism signaling
signaling
response to wounding
multicellular organismal process
ciliary or flagellar motility
cell communication
1.7
1.6
1.6
1.5
1.5
1.9
1.6
10.2
1.5
0.0
0.5
1.0
1.5
2.0
−log10(FDR)

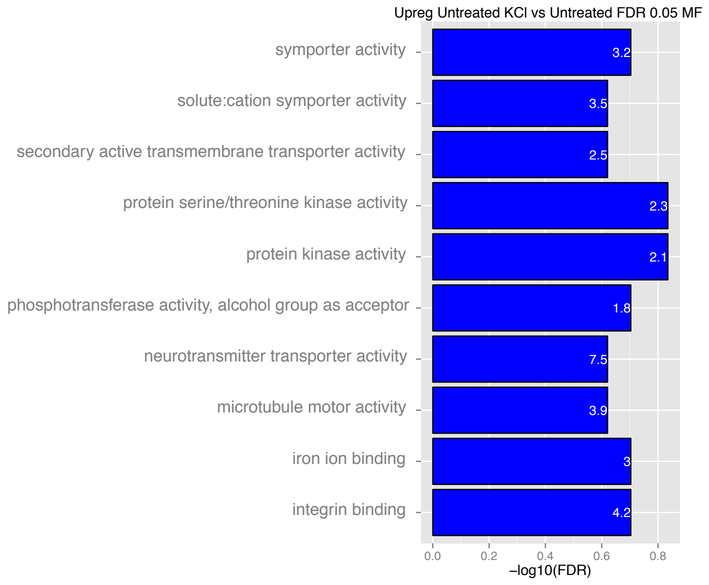
Upreg Untreated KCl vs Untreated FDR 0.05 MF
symporter activity
solute:cation symporter activity
secondary active transmembrane transporter activity
protein serine/threonine kinase activity
protein kinase activity
phosphotransferase activity, alcohol group as acceptor
neurotransmitter transporter activity
microtubule motor activity
iron ion binding
integrin binding
3.2
3.5
2.5
2.3
2.1
1.8
7.5
3.9
3
4.2
0.0
0.2
0.4
0.6
0.8
−log10(FDR)

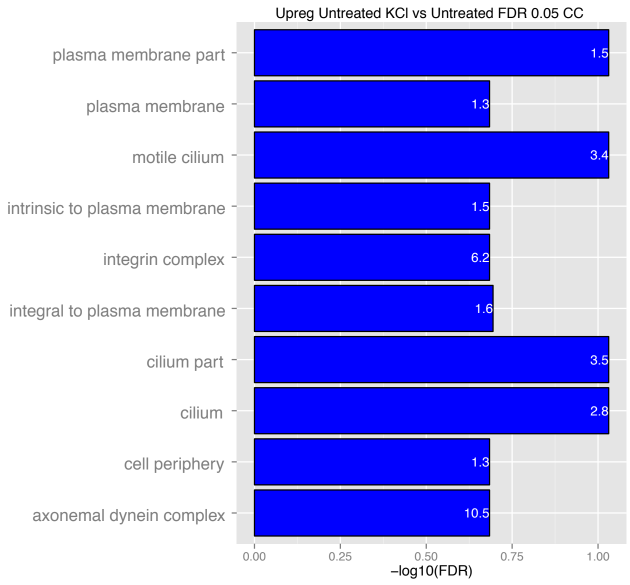
Upreg Untreated KCl vs Untreated FDR 0.05 CC
plasma membrane part
plasma membrane
motile cilium
intrinsic to plasma membrane
integrin complex
integral to plasma membrane
cilium part
cilium
cell periphery
axonemal dynein complex
1.5
1.3
3.4
1.5
6.2
1.6
3.5
2.8
1.3
10.5
0.00
0.25
0.50
0.75
1.00
−log10(FDR)

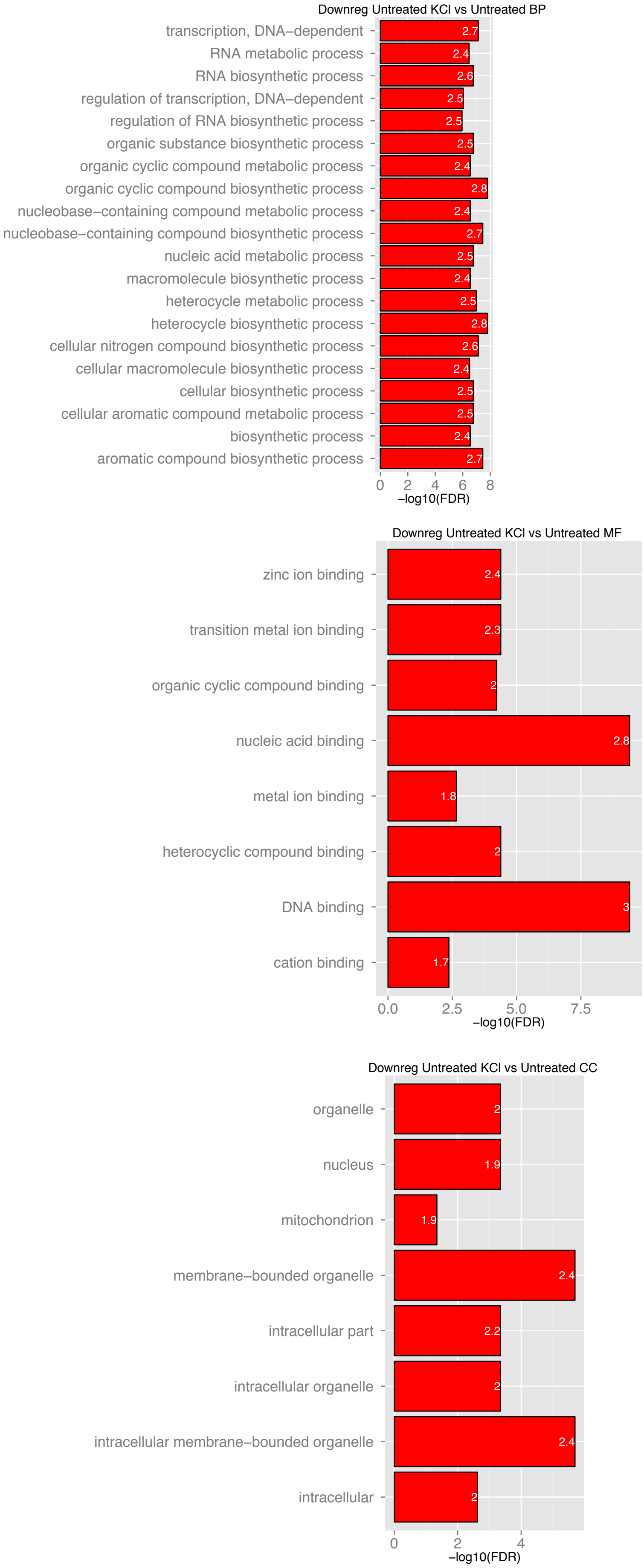

Supplementary Fig. 9: Gene ontology enrichment terms for deep sequencing results from control untreated iPSC-derived neurons as compared with activated control iPSC-derived neurons (Blue:

GO analysis for significantly upregulated genes; Red: GO analysis for significantly downregulated genes).

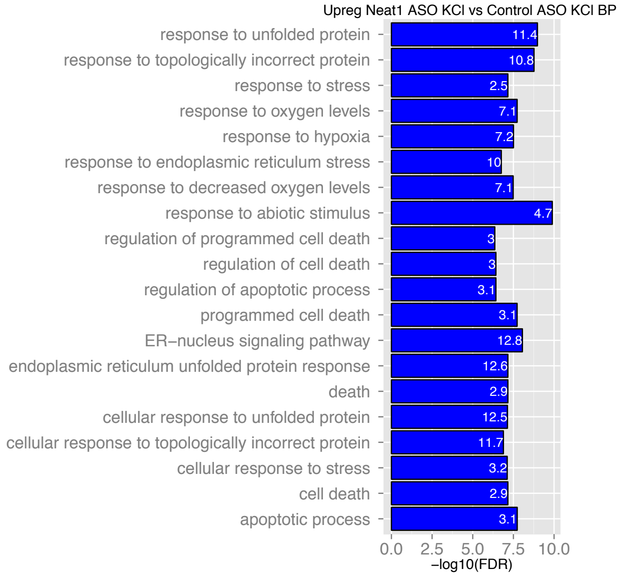

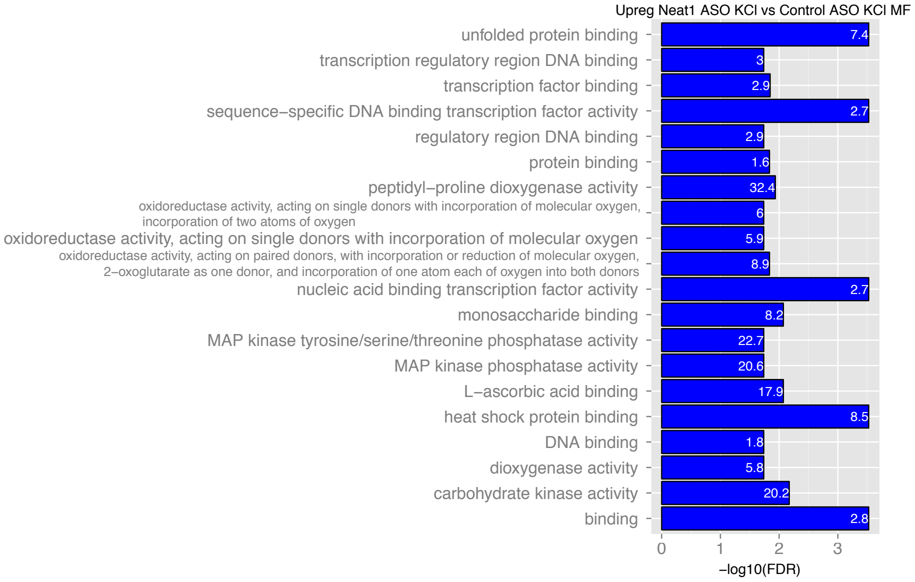

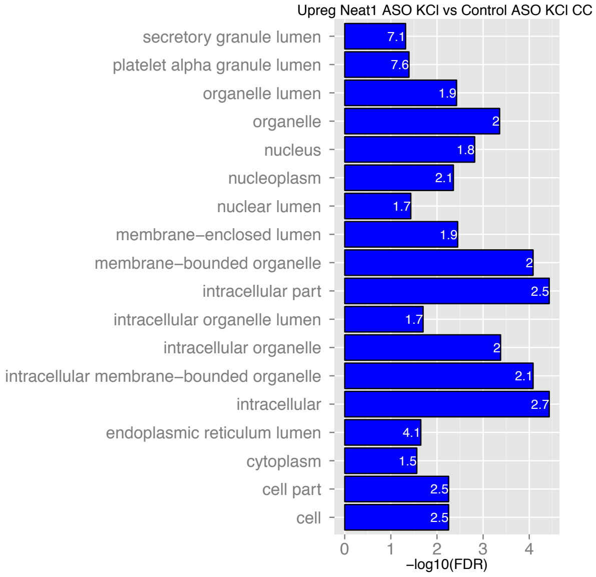

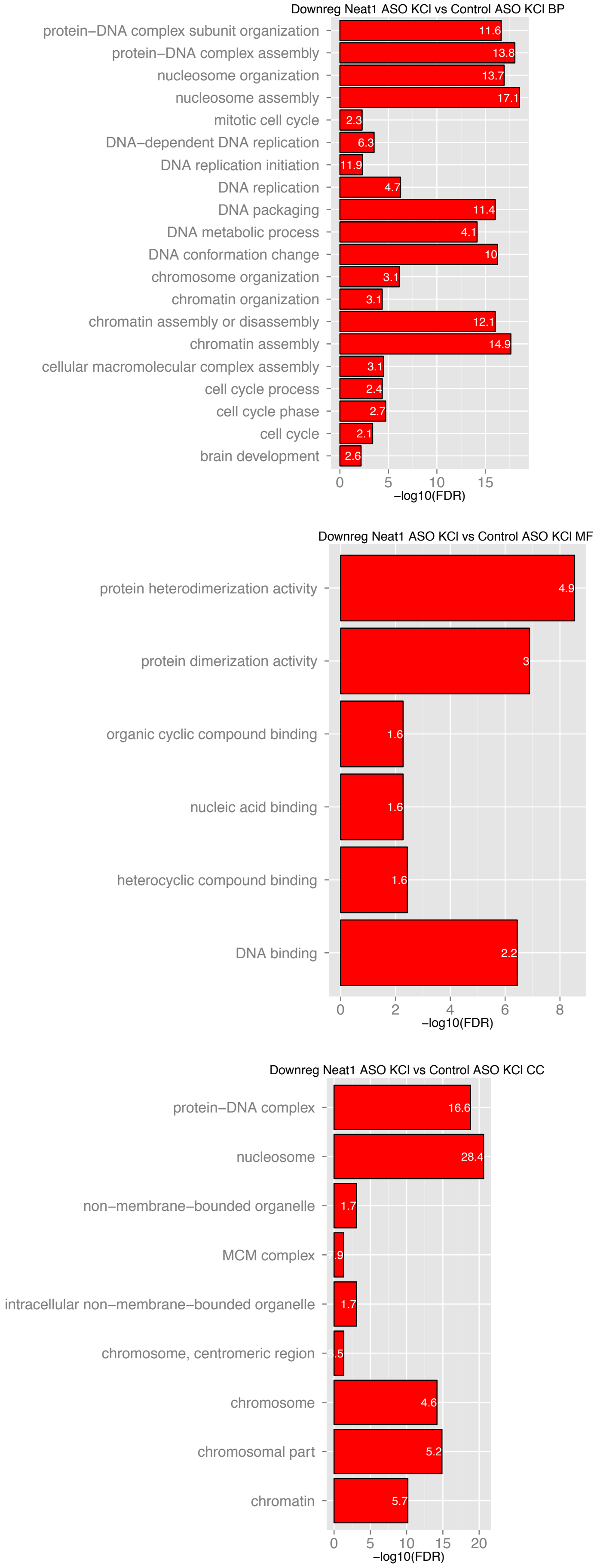

Supplementary Fig. 10: Gene ontology enrichment terms for deep sequencing results from activated control ASO-treated iPSC-derived neurons as compared with activated NEAT1 ASO

iPSC-derived neurons (Blue: GO analysis for significantly upregulated genes; Red: GO analysis for significantly downregulated genes).

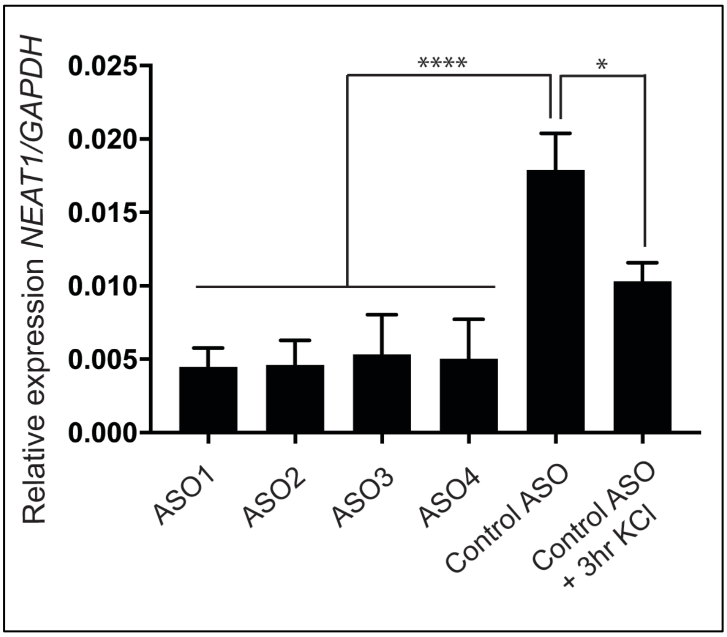

Supplementary Fig. 11: ASOs directed at NEAT1 significantly reduce NEAT1 transcript as compared with a scrambled control ASO. The control ASO does not impede NEAT1 downregulation following 3 hours of KCl activation. (n=3; One-way ANOVA test with a Tukey's multiple comparison post hoc test *p value< 0.05; ****p value< 0.0001).

Supplementary Table 1

Z scores: Protein microarray - NEAT1

SymbolName	ChipID	Neat1-1 (z-score)	Neat1-2 (z-score)
-	H1	9.326	9.11
-	IOH11952	8.777	0
-	IOH23182	4.979	0
-	NME2	18.956	65.62
-	a-Hum6	0	5.262
-	p53	12.587	-0.527
AADAT	IOH22548	1.454	6.598
ACOX1	IOH27813	3.038	4.032
ADH4	IOH12335	3.692	0.31
ALDH1A1	IOH57094	2.325	10.372
ASMTL	IOH3971	7.772	0
BRPF1	IOH29001	8.267	0
C14orf179	IOH12238	5.387	0
C22orf9	IOH28970	4.306	0
CAT	IOH41295	4.317	0
CES2	IOH41749	5.833	0
CHRNA4	IOH63277	4.612	0
CNOT8	IOH27767	6.861	0
CRABP1	IOH10831	4.554	0
DCTD	IOH54732	1.75	9.159
DNAJB5	IOH57560	13.909	10.733
EVL	IOH21728	9.381	6.046
F2	IOH29572	2.609	11.27
FABP7	IOH11228	4.204	0
GABPA	IOH27140	5.511	0
GAS2	IOH27961	5.447	0
GPI	IOH5104	4.976	0
GPR119	IOH28379	7.023	0
GPR161	IOH11641	6.864	0
GSTA1	IOH28934	7.562	15.494
GSTA2	IOH5711	1.977	8.395
GSTA3	IOH12054	13.073	0
GSTO1	IOH4381	0	6.915
HCFC1R1	IOH40008	7.021	0
HOXA5	IOH10527	6.574	0
HPGD	IOH14612	6.555	0
HSN2	IOH57869	7.066	0
HSPA4	IOH4058	4.409	0
HSPC142	IOH4609	4.558	0
IL18BP	IOH45456	14.55	0

IL1F6	IOH35127	0	4.464
KCNAB1	IOH25842	7.604	20.486
KCNAB1	IOH29846	0.714	11.611
KCNAB2	IOH29643	1.925	44.943
KCNIP1	IOH26654	4.908	0
KIF7	IOH63579	7.622	0
Kua	IOH59436	4.18	0
MAGEB10	IOH63565	4.308	0
MB	IOH12916	1.759	8.088
MCM7	IOH12526	4.425	0
MGC10981	IOH5578	6.737	0
MGC34800	IOH22740	1.573	4.911
MGC35030	IOH27440	2.788	6.184
MGP	IOH63180	6.157	0
NAT1	IOH26700	8.128	0
NPM1	IOH27884	5.628	0
NUAK2	IOH21129	7.644	0
PCNP	IOH56032	3.761	2.751
PIN1	IOH5702	3.567	2.534
PRMT6	IOH5353	5.064	0
PSAT1	IOH4946	2.254	8.604
PYDC1	IOH42416	2.857	2.554
QDPR	IOH4152	47.71	55.502
RAB33B	IOH62147	4.819	0
RABEP2	IOH62067	5.802	0
RAD23A	IOH62598	0	4.5
RAD23B	IOH9971	17.179	9.681
RHEB	IOH14352	4.033	0
RNH1	IOH25734	8.341	0
RP11-321G1.2	IOH44466	2.195	1.878
SCGN	IOH56717	1.837	4.599
SCP2	IOH7548	1.675	4.472
SEC13	IOH5238	5.026	0
SPG21	IOH4511	0	9.188
SSBP2	IOH10629	4.083	0
TAGLN	IOH5527	10.76	13.094
TBCD	IOH29351	6.096	0
TGM4	IOH7198	5.151	2.498
TGOLN2	IOH7570	5.991	0
TIMP1	IOH7468	3.897	0.47
TMOD4	IOH12948	5.722	0
TNNT2	IOH5297	14.441	15.334
TPI1	IOH27928	6.009	0
TPMT	IOH7206	5.667	4.044
TXNRD1	IOH52112	0	7.881
UBE2C	IOH10068	-0.208	4.517

UBE2V2	IOH14681	5.064	0
UGT3A2	IOH63460	4.373	0
VGLL4	IOH29305	8.232	0
YKT6	IOH5843	8.981	0
ZADH2	IOH21771	6.943	0
ZNF385	IOH22141	0	8.29

Supplementary Table 2

Primer sequences used for investigating transcript levels in iPSC-related differentiation and mature neurons.

Target Gene	Forward Primer (5'-3')	Reverse Primer (3'-5')
PAX6	CAGCACCAGTGTCTACCAACCA	CAGATGTGAAGGAGGAAACCG
NEAT1	TCGGGTATGCTGTTGTGAAA	TGACGTAACAGAATTAGTTCTTACCA
TBR1	TAACAATGGGCAGATGGTGG	AGGGAAAGTGAACGTCTGCA
TBR2	ACAATAACATGCAGGGCAACA	TTGCGCCTTTGTTATTGGTGAG
GOMAFU	GTGTGTGTCTGCTGAGGTG	CTGGGGTTAGTAAGAAGAGAA
DISC1	AAGCAGCAGCTACAGAA	CTGGACTTGCTGCTCTTGC
GAPDH	GTGAACCATGAGAAGTATGACAAC	CATGAGTCCTTCCACGATACC
7SL	ATCGGGTGTCCGCACTAAGTT	CAGCACGGGAGTTTTGACCT
MASH1	TCTCATCCTACTCGTCGGACGA	CTGCTTCCAAAGTCCATTCGCAC
BDNF	CTCCGCCATGCAATTTCCAC	GCCTTCATGCAACCAAAGTA
ARC	GCGGCTCTGAGGAGTACTGGCT	ATGGCCTCTCGGGACAGCGT
FOS	CCGGAGGAGGGAGCTGACTGA	GGATCTTGCAGGCAGGTCGGTG
EGR1	AGCCCTACGAGCACCTGA	GGCAGTCGAGTGGTTTGG
SYP	ATCTTCGCCTTTGCCACATG	TACACTTGGTGCAGCCTGAA
GRIA2	AAGGAAAAGACCAGTGCCCTCAGTC	AGCATTGCCAAACCAAGGCCCC

Supplementary Table 3: Leading edge gene analysis (Neat1 ASO KCl versus Control ASO KCl)

Gene set	Gene	Fold change	Enrichment score
Cation channel activity	CHRNB4	1.539355397	0.061891958
	SCN9A	1.349445105	0.117198445
	SLC4A11	0.973863184	0.13591725
	CACNG4	0.92418772	0.17197502
	TRPC3	0.867695749	0.20322531
	CACNA1A	0.800219119	0.22888248
	KCNIP2	0.781355321	0.2599465
	KCNC3	0.746328533	0.28473666
	KCNA2	0.706319571	0.30553043
	CACNA1C	0.624155939	0.30757207
	CHRNA4	0.623011649	0.3350074
	CACNA1B	0.605372488	0.35488778
	SCNN1D	0.580967188	0.37146994
	CACNB3	0.552327037	0.38478503
	KCNJ1	0.536561787	0.40121877
	RYR2	0.530061007	0.42140636
	KCNB2	0.504055262	0.430723
	SCN11A	0.494159609	0.44908357
	CACNA1E	0.485940903	0.4667822
	KCNA3	0.462315798	0.47481957
	KCNK10	0.431241781	0.4770541
	CHRNB2	0.425453842	0.49278167
	CACNA1H	0.399637669	0.49352854
	RYR3	0.381126195	0.49947742
Gated channel activity	CHRNB4	1.539355397	0.061059184
	SCN9A	1.349445105	0.11563587
	CACNG4	0.92418772	0.12625779
	TRPC3	0.867695749	0.15703788
	CACNA1A	0.800219119	0.18226069
	CLCN1	0.799360096	0.21737565
	KCNIP2	0.781355321	0.24831112
	KCNC3	0.746328533	0.2726964
	KCNA2	0.706319571	0.29310638
	CNGA3	0.629976094	0.29704857
	CACNA1C	0.624155939	0.32272685
	CHRNA4	0.623011649	0.34982568
	CACNA1B	0.605372488	0.36937755
	SCNN1D	0.580967188	0.38564393
	CACNB3	0.552327037	0.39865825
	KCNJ1	0.536561787	0.41480058
	RYR2	0.530061007	0.43470117
	KCNB2	0.504055262	0.44374272
	SCN11A	0.494159609	0.46183562

	CACNA1E	0.485940903	0.479271
	KCNA3	0.462315798	0.4870559
	CHRNB2	0.425453842	0.48541436
	CACNA1H	0.399637669	0.48594165
	RYR3	0.381126195	0.49168226
Voltage Gated Chanel Activity	SCN9A	1.349445105	0.079638876
	CACNG4	0.92418772	0.112296544
	CACNA1A	0.800219119	0.14893588
	CLCN1	0.799360096	0.20306839
	KCNIP2	0.781355321	0.25259852
	KCNC3	0.746328533	0.29475325
	KCNA2	0.706319571	0.3319844
	CACNA1C	0.624155939	0.3485741
	CACNA1B	0.605372488	0.38202572
	CACNB3	0.552327037	0.39872515
	KCNJ1	0.536561787	0.42764482
	KCNB2	0.504055262	0.44510105
	SCN11A	0.494159609	0.47495633
	CACNA1E	0.485940903	0.503959
	KCNA3	0.462315798	0.5227631
	SCN9A	1.349445105	0.08883411
	CACNG4	0.92418772	0.1277957
	CACNA1A	0.800219119	0.16989088
	KCNIP2	0.781355321	0.22445057
	KCNC3	0.746328533	0.27169138
	KCNA2	0.706319571	0.3137368
	CACNA1C	0.624155939	0.3345855
	CACNA1B	0.605372488	0.37216282
	CACNB3	0.552327037	0.3926305
	KCNJ1	0.536561787	0.42520714
	KCNB2	0.504055262	0.44610167
	SCN11A	0.494159609	0.47932398
	CACNA1E	0.485940903	0.5116378
	KCNA3	0.462315798	0.53359467
	CACNA1H	0.399637669	0.52590126
	KCNC1	0.329108179	0.50448895
	KCNH2	0.302116901	0.50991
	CACNB1	0.282588333	0.5162929
	KCNQ3	0.281389356	0.53624153
	KCNH1	0.251571983	0.5328158
	CACNA1G	0.199691683	0.5042918
	PKD2	0.196194023	0.51566476
	KCNQ2	0.19246687	0.5273462
	KCNC4	0.191897437	0.54140776
	CACNA2D1	0.17911005	0.5441611
Glutamate Receptor Activity	GRM5	1.674967051	0.14774728

	GRIN2A	1.113351941	0.22934513
	GRM1	0.921867371	0.29512927
	GRIK1	0.86959666	0.36737815
	GABBR2	0.811231852	0.43277484
	GRIK5	0.793440402	0.5013932
	GRM7	0.709056318	0.5449486
	GRIK3	0.6955145	0.6045811
Substrate Specific Channel Activity	CHRNB4	1.539355397	0.0508836
	SCN9A	1.349445105	0.09654061
	SLC4A11	0.973863184	0.10828
	CACNG4	0.92418772	0.13772784
	TRPC3	0.867695749	0.16277033
	CACNA1A	0.800219119	0.18270011
	CLCN1	0.799360096	0.21253283
	KCNIP2	0.781355321	0.23830327
	KCNC3	0.746328533	0.25775242
	KCNA2	0.706319571	0.2734895
	CNGA3	0.629976094	0.2732565
	CACNA1C	0.624155939	0.29480943
	CHRNA4	0.623011649	0.3177912
	CACNA1B	0.605372488	0.3333391
	SCNN1D	0.580967188	0.34576163
	CACNB3	0.552327037	0.3551203
	KCNJ1	0.536561787	0.36771318
	RYR2	0.530061007	0.38410932
	KCNB2	0.504055262	0.38981327
	SCN11A	0.494159609	0.40463886
	CACNA1E	0.485940903	0.41886106
	PDPN	0.478793919	0.43318254
	KCNA3	0.462315798	0.4416593
	KCNK10	0.431241781	0.4407988
	CHRNB2	0.425453842	0.4534828
	CACNA1H	0.399637669	0.45136046
Calcium Channel Activity	CACNG4	0.92418772	0.07997219
	TRPC3	0.867695749	0.18723749
	CACNA1A	0.800219119	0.28300643
	CACNA1C	0.624155939	0.3163076
	CACNA1B	0.605372488	0.38871345
	CACNB3	0.552327037	0.44097003
	RYR2	0.530061007	0.49996254
	CACNA1E	0.485940903	0.54314095
	CACNA1H	0.399637669	0.5457785
	RYR3	0.381126195	0.5851361
Ion Channel Activity	CHRNB4	1.539355397	0.051945042
	SCN9A	1.349445105	0.09853246

	SLC4A11	0.973863184	0.11094487
	CACNG4	0.92418772	0.14103006
	TRPC3	0.867695749	0.16667113
	CACNA1A	0.800219119	0.18715316
	CLCN1	0.799360096	0.21753682
	KCNIP2	0.781355321	0.24384604
	KCNC3	0.746328533	0.2638102
	KCNA2	0.706319571	0.28003487
	CNGA3	0.629976094	0.28023782
	CACNA1C	0.624155939	0.30222103
	CHRNA4	0.623011649	0.3256322
	CACNA1B	0.605372488	0.34159788
	SCNN1D	0.580967188	0.3544215
	CACNB3	0.552327037	0.3641617
	KCNJ1	0.536561787	0.3771249
	RYR2	0.530061007	0.39388666
	KCNB2	0.504055262	0.39993897
	SCN11A	0.494159609	0.4151054
	CACNA1E	0.485940903	0.42966282
	KCNA3	0.462315798	0.43470582
	KCNK10	0.431241781	0.43414375
	CHRNB2	0.425453842	0.44712126
	CACNA1H	0.399637669	0.44527563
	RYR3	0.381126195	0.44875553
Chromatin Binding	CHAF1A	1.468409538	0.11113943
	CHAF1B	1.328212261	0.21420008
	POLE	1.090634942	0.2894578
	CENPA	0.761529207	0.30907512
	SUV39H1	0.737754464	0.36273086
	NCOA6	0.634977043	0.3835252
	CDCA5	0.581609964	0.4100434
	RCC1	0.574044108	0.45341778
	POLA1	0.464256197	0.43847722
	POLD1	0.455369651	0.4705286
	TOP2A	0.446499437	0.5023737
	SMC1A	0.399125367	0.5051947
	ACTL6A	0.385815501	0.5291035
Rhodopsin Like Receptor Activity	OPRK1	1.392905474	0.09667229
	HTR2A	1.342810988	0.1988003
	TAS2R7	1.208271027	0.28519136
	TAS2R9	1.064439893	0.35813758
	CHRM2	0.995953262	0.42851558
	TAS2R8	0.864032805	0.4795425
	SSTR2	0.784535825	0.5275187
	CNR1	0.628129244	0.5311371

Phosphotransferase Activity	NME5	1.066567183	0.10925082
	AK5	0.901457846	0.20834263
	DTYMK	0.820766985	0.30340213
	PMVK	0.800252199	0.40329456
	MPP1	0.669306636	0.45468205
	NME6	0.616903484	0.5166878
	NME4	0.595617175	0.5858471
Metal Ion Transmembrane transporter activity	CHRNB4	1.539355397	0.04892613
	SCN9A	1.349445105	0.09286778
	SLC4A11	0.973863184	0.10335674
	CACNG4	0.92418772	0.13162872
	TRPC3	0.867695749	0.1555657
	CACNA1A	0.800219119	0.17447406
	KCNIP2	0.781355321	0.19895634
	KCNC3	0.746328533	0.21745335
	KCNA2	0.706319571	0.2322878
	CACNA1C	0.624155939	0.22904167
	CHRNA4	0.623011649	0.2512326
	CACNA1B	0.605372488	0.26600808
	SCNN1D	0.580967188	0.27768785
	CACNB3	0.552327037	0.28633893
	ATP2A1	0.546734989	0.30332884
	KCNJ1	0.536561787	0.3181819
	RYR2	0.530061007	0.33390334
	ABCC8	0.520202339	0.3480148
	KCNB2	0.504055262	0.35793144
	SCN11A	0.494159609	0.37212774
	CACNA1E	0.485940903	0.38573092
	KCNA3	0.462315798	0.38986027
	KCNK10	0.431241781	0.38844246
	CHRNB2	0.425453842	0.40058467
	CACNA1H	0.399637669	0.3979451
	FXN	0.390103638	0.4062994
	RYR3	0.381126195	0.4149167
	ITPR2	0.362534702	0.41571572
	SLC40A1	0.343906641	0.4155435
	KCNC1	0.329108179	0.4177055
	KCNH2	0.302116901	0.4114496
	KCNN3	0.286589831	0.4098579
	CACNB1	0.282588333	0.41739917
	KCNQ3	0.281389356	0.42651692
Nucleotide Kinase Activity	TK1	1.821220517	0.17421971
	NME5	1.066567183	0.25281298
	AK5	0.901457846	0.32333001
	DTYMK	0.820766985	0.392375
	MPP1	0.669306636	0.4190866
	NME6	0.616903484	0.46153453

	NME4	0.595617175	0.51181555
	TK2	0.525185645	0.53154033
	PNKP	0.44220084	0.532694
Micortubule Motor Activity	KIF11	1.029611945	0.1414695
	KIF4A	0.918430984	0.284693
	CENPE	0.723258138	0.3706619
	KIF22	0.709087014	0.48614517
	KIFC3	0.41476658	0.4275246
	KIF2C	0.396221399	0.4824519
	KIF23	0.328601599	0.49344435
	BBS4	0.29363215	0.5193793
	KIF3B	0.283915132	0.5603987
	KIF1B	0.241681084	0.5677672
Integrin Binding	ADAM11	1.204151988	0.12545028
	ADAMTS5	1.008517265	0.23272742
	THY1	0.731645763	0.27758908
	COL16A1	0.632058859	0.3235298
	TNXB	0.630646467	0.39907917
	ADAMTS8	0.611449718	0.46513203
	ADAMTS13	0.6107409	0.5382875
